# Downregulated miR-18b-5p triggers apoptosis by inhibition of calcium signaling and neuronal cell differentiation in transgenic SOD1 (G93A) mice and SOD1 (G17S and G86S) ALS patients

**DOI:** 10.1186/s40035-020-00203-4

**Published:** 2020-07-01

**Authors:** Ki Yoon Kim, Yu Ri Kim, Kyung Won Choi, Mijung Lee, Somyung Lee, Wooseok Im, Je-Young Shin, Jin Young Kim, Yoon Ho Hong, Manho Kim, Jong-Il Kim, Jung-Joon Sung

**Affiliations:** 1 Department of Neurology, Seoul National University Hospital 28 yongon-Dong, Chongno-gu Seoul, 110-744 Republic of Korea; 2 Division of Mass Spectrometry Research, Korea Basic Science Institute, Daejun, South Korea; 3 Department of Neurology, Seoul National University Seoul Metropolitan Government Boramae Medical Center, Seoul, South Korea; 4 Department of Biochemistry and Molecular Biology, Seoul National University College of Medicine, Seoul, South Korea

**Keywords:** miRNAs, Hif1α, Mef2c, Mctp1 and Rarb

## Abstract

**Background:**

MicroRNAs (miRNAs) are endogenous non-coding RNAs that regulate gene expression at the post-transcriptional level and are key modulators in neurodegenerative diseases. Overexpressed miRNAs play an important role in ALS; however, the pathogenic mechanisms of deregulated miRNAs are still unclear.

**Methods:**

We aimed to assess the dysfunction of RNAs or miRNAs in fALS (SOD1 mutations). We compared the RNA-seq of subcellular fractions in NSC-34 WT (hSOD1) and MT (hSOD1 (G93A)) cells to find altered RNAs or miRNAs. We identified that Hif1α and Mef2c were upregulated, and Mctp1 and Rarb were downregulated in the cytoplasm of NSC-34 MT cells.

**Results:**

SOD1 mutations decreased the level of miR-18b-5p. Induced Hif1α which is the target for miR-18b increased Mef2c expression as a transcription factor. Mef2c upregulated miR-206 as a transcription factor. Inhibition of Mctp1 and Rarb which are targets of miR-206 induces intracellular Ca^2+^ levels and reduces cell differentiation, respectively. We confirmed that miR-18b-5p pathway was also observed in G93A Tg, fALS (G86S) patient, and iPSC-derived motor neurons from fALS (G17S) patient.

**Conclusions:**

Our data indicate that SOD1 mutation decreases miR-18b-5p, which sequentially regulates Hif1α, Mef2c, miR-206, Mctp1 and Rarb in fALS-linked SOD1 mutation. These results provide new insights into the downregulation of miR-18b-5p dependent pathogenic mechanisms of ALS.

## Background

Amyotrophic lateral sclerosis (ALS) is a neurodegenerative disorder that causes the degeneration of upper and lower motor neurons (MNs) in the spinal cord, brainstem, and cerebral cortex [1–5]. The most pathogenic mechanisms of ALS are gene mutations in the following genes: Cu/Zn superoxide dismutase 1 (SOD1), Fused in Sarcoma (FUS), C9orf72 and TAR DNA-binding protein 43 (TDP-43) [5–12]. These gene mutations are related to various RNA metabolisms [2, 5–12]. Current findings strikingly suggest that gene mutations regulate the biogenesis of microRNAs (miRNAs); these miRNAs play a pivotal role in the pathogenesis of neurodegenerative diseases [9, 10]. However, the specific interactive mechanisms of gene mutations and miRNAs related to ALS have not been fully elucidated [11, 12].

RNA biogenesis, including mRNA transcription, splicing, export, stability, and microRNAs (miRNAS), is emerging as an important factor in the pathogenesis of ALS [1, 13]. Recently, differentially expressed miRNAs (miR-23a, miR-455, miRb1336 and miR-b2403) have been identified between normal and diseased tissue [9, 14], and miRNAs are presently emerging as key factors in ALS, as well as in Huntington’s disease (miR-9), Alzheimer’s disease (miR29a/b-1), and Parkinson’s disease (miR-205) [9, 15–22]. miRNAs are small non-coding single-stranded RNA molecules that regulate protein synthesis via RNA-dependent post-transcriptional gene regulation [23–25]. According to recent reports, miRNAs are associated with cellular processes, such as calcium signaling, and neuronal differentiation [26–30].

Calcium signaling is a ubiquitous system that is involved in the regulation of cellular processes such as cell proliferation and apoptosis [29, 30]. Intracellular Ca^2+^ levels are tightly controlled by transporters, and binding proteins [30]. The multiple-C2 domain transmembrane protein 1 (Mctp1), which has Ca^2+^ binding-affinity C2 domains, is essential for neuronal calcium signaling [30, 31]. High cellular Ca^2+^ concentration leads to apoptosis through mitochondrial dysfunction [29]. According to a recent report, intracellular Ca^2+^ levels are not only increased, but Ca^2+^ buffering is also perturbed following SOD1 mutation in ALS [32].

Cell growth and differentiation are regulated by retinoids (vitamin A derivatives) and play a prominent role in neuronal cells [33]. Retinoic acid (RA) is a biologically active form of vitamin A and it regulates cell proliferation and differentiation [33]. During this process, retinoic acid receptor beta (Rarb), a transcriptional co-regulator with retinoic X receptor (RXR) mediates RA response [34]. Overexpression of mutated human SOD1 in MNs, such as NSC-34 cells, proves impaired cell differentiation and induced apoptosis [35, 36]. Dysregulation of calcium signaling and neuronal cell differentiation are related to apoptosis and are representative events in ALS pathogenesis [36–40].

In this study, we performed whole transcriptome analysis to explain the role of SOD1 mutation by studying the subcellular fractionation of NSC-34 hSOD1 (wtNSC-34) and hSOD1 (G93A) (mtNSC-34) cells. With respect to our RNA-seq results, we found several altered RNAs [hypoxia inducible factor 1 alpha (Hif1α), myocyte specific enhancer factor 2c (Mef2c), Mctp1, and Rarb] in mtNSC-34 cells. The RNA levels of Hif1α and Mef2c were upregulated in the nucleus and the cytoplasm of mtNSC-34 cells. Specifically, the cytoplasmic RNAs of Hif1α and Mef2c were higher in number than nuclear RNAs in mtNSC-34 cells. Furthermore, Mctp1 and Rarb transcripts were highly expressed in the nucleus, but were decreased in the cytoplasm of mtNSC-34 cells. For the reason that Hif1α, Mef2c, Mctp1, and Rarb were observed to be different in the cytoplasm of mtNSC-34 cells, we hypothesized that these genes were post-transcriptionally regulated in mtNSC-34 cells. To identify the post-transcriptional regulation of Hif1α, Mef2c, Mctp1, and Rarb, we found that miR-18b-5p was involved in the regulation of Hif1α, and miR-206 regulated both Mctp1 and Rarb. To determine whether or not miR-18b-5p is related to SOD1 mutation in ALS, we validated the expression of miR-18b-5p, miR-206, Hif1α, Mef2c, Mctp1, and Rarb in vitro and in vivo. Our results indicate that a new molecular pathway for miR-18b-5p, which sequentially regulates Hif1α, Mef2c, miR-206, Mctp1, and Rarb is involved in the pathogenic mechanisms of ALS-linked SOD1 mutations.

## Methods

### Animals

Animal studies were carried out in accordance with the Institutional Animal Care and Use Committee (IACUC) guidelines of Seoul National University for the care and use of laboratory animals. Transgenic mice expressing the human G93A-mutated SOD1 gene (B6SJL-Tg (SOD1-G93A) 1 Gur/J; Jackson Laboratory, Bar Harbor, Me, USA) were used in this study. WT and SOD1-G93A mice used for biochemical analyses were sacrificed 120 days after birth.

### Preparation of NSC for in vitro model cell

Neural stem cells (NSC) were derived from the subventricular zone of 9-week-old mice. The culture method has been used previously [41]. Briefly, mice brain tissues were dissected and minced in a dish containing HBSS. The cells were trypsinized with TripleExpress (12604–013, Thermo Scientific, Waltham MA USA) and incubated for 15 min at 37 °C. And then cells were seeded in a 6-well plate after centrifuging and resuspending with DMEM/F12 (11,320,033, Invitrogen, Waltham MA USA) supplied with 1% PSA (penicillin-streptomycin; 15,140,122, Invitrogen, Carlsbad CA USA), 2% B27 Supplement (17,504,044, Gibco, BRL, Carlsbad CA USA), 10 ng/mL epidermal growth factor (EGF; Invitrogen, Carlsbad CA USA), and 10 ng/mL basic fibroblast growth factor (CTP0261, bFGF; Invitrogen, Carlsbad CA USA) for culturing NSCs. For inducing differentiation [42], when the cells formed neurospheres sized about 50–100 μm in diameter, they were resuspended and transferred into a sterile 15-ml tube. The neurosphere pellet was obtained by centrifuging at 100×g for 5 min at room temperature, and resuspended with differentiation culture medium (DMEM/F12, 1% PSA, 2% B27, and 5% FBS (FBS;12,483–020, Gibco, Grand Island NY USA)).

### Annexin V and PI analysis by flowcytometry

NSCs were seeded in 6-well tissue culture plates. For using Annexin-V-FITC and PI Apoptosis Detection Kit (556,547, BD Bioscience, Eugene, NJ, USA), the adherent NSCs were detached with TripleExpress (12605–010, GIBCO, NY 14072 USA). The culture medium was then added to inactivate trypsin. The supernatant was removed after centrifuging for 5 min at 1500×g. and cells were stained with Annexin V-FITC and PI according to the manufacturer’s instructions. The cells were analyzed immediately after staining using a FACSCalibur (BD Biosciences, San Jose, CA). For each measurement, at least 20,000 cells were counted. Fluorescence was evaluated using the green or red channel, and the data were analyzed using Flowwing Software (Version 2.5.1, Unversity of Turku, Filand).

### RNA interference experiments and Western blot analysis

40 nM of siRNA duplex were transfected in NSC-34 cells with RNAiMax transfection reagent (13778–150, Invitrogen, Carlsbad CA USA) according to the manufacturer’s instructions. miR-18b-5p and miR-206 inhibitor (anit-18b and anti-206) were obtained from COSMO GENETECH. siRNAs were synthesis from COSMO GENETECH. Target sequences of mouse siHif1α, siMctp1 and siRarb were 5′-AAGCAUUUCUCUCAUUUCCUCAUGG-3′ 5′-GCCACUAUAUAUCAAGGUATT-3′ and 5′-GGAGCCUUCAAAGCAGGAATT-3′. NSC-34 cells were collected at 48 h after siRNA transfection. NSC-34 cells were collected at 48 h after anti-18b, miR-206, mCherry-Mctp1 and eGFP-Rarb transfection with Lipofectamine 2000 (11,668,019, Invitrogen, Carlsbad CA USA). The cells were dispersed by pipetting and 20–30 mg of frozen tissues were homogenized in lysis buffer (10 mM Tris at pH 7.4, 1 mM ethylenediaminetetra acetic acid [EDTA] at pH 8.0500 mM NaCl, and 0.5% Triton X-100) and incubated for 30 min on ice. The samples were centrifuged at 15000 rpm at 4 °C for 20 min, obtaining a supernatant (soluble proteins) and a pellet. The pellets was re-suspended in lysis buffer and sonicated 3 times for 10 s and shaken for 1 h at 4 °C. Samples were centrifuged at 15000 rpm at 4 °C for 20 min, obtaining a supernatant (insoluble proteins). (Primary antibodies used in this study are mouse anti-Hif1α antibody (NB100–449, NOVUS, Abingdon OX14 3NB UK), rabbit anti-Mef2c antibody (LS-C31031, LSBio, Seattle WA USA), rabbit anti-Mctp1 antibody (NBP1–83604, Novus Biologicals, Oakville ON L6M 2 V5 Canada), rabbit anti-Rarb (ab53161, abcam, Cambridge CB2 0AX UK), rabbit anti-Bax (SC-493, Santa Cruz, Dallas Texas USA), rabbit anti-Bcl2 (SC-492, Santa Cruz, Dallas Texas USA), mouse anti-Flag (F3165, SIGMA, Burlington MA USA), goat anti-GFP antibody (600–101-215, Rockland, Gilbertsville PA USA), rabbit anti-mCherry (M11217, Thermofisher, Waltham MA USA) and mouse anti β-actin (sc-47,778, SantaCruz, Dallas Texas USA), rabbit anti-SOD1 (97,959, abcam, Cambridge CB2 0AX UK).

### Intracellular Ca^2+^ assay

The day before the experiment, plate the cells overnight in growth medium using 4 × 10^4^ to 8 × 10^4^ cells per well at a plating volume of 100 μl per well for 96-well plates. After 48 h, FLUOFORTE Dye-Loading Solution added to each well and incubated the cell plates for 45 min at 37 °C and 15 min at room temperature. Then, fluorescence was measured at 490/525 nm using a fluorescence plate reader. The changes in intracellular calcium levels of each group were measured by quantifying the fold change of the fluorescence level of Fluo-4 with respect to the basal level.

### Lactate dehydrogenase (LDH) release assay

Cell culture medium was collected and briefly centrifuged. The supernatants were transferred into wells in 96-well plates. Equal amounts of lactate dehydrogenase assay substrate (MAK066, SIGMA, Burlington MA USA), enzyme and dye solution were mixed. A Half volume of the above mixture was added to one volume of medium supernatant. After incubating at room temperature for 30 min, the reaction was terminated by the addition of 1/10 volume of 1 N HCl to each well. Spectrophotometrical absorbance was measured at a wavelength of 490 nm and reference wavelength of 690 nm.

### Plasmid construction

The 3’UTR of mouse Mctp1 was amplified by PCR from NSC-34 cDNA (forward primer, 5′- CCGCTCGAGAAAGCTTGAATAATAGAAAT-3′ and reverse primer, 5′- CTAGTCTAGAATACATGGGTTTTTTGTTTG-3′). The 3’UTR of mouse Rarb was amplified by PCR from NSC-34 cDNA (forward primer, 5′- CCGCTCGAGAACGTGTAATTACCTTGAAA-3′ and reverse primer, 5′- CTAGTCTAGACAAAGTCTTCAGAAACTTAA-3′). The 3’UTR of mouse Hif1α was amplified by PCR from NSC-34 cDNA (forward primer, 5′- CCGCTCGAGTGTTGGTTATTTTTGGACACT-3′ and reverse primer, 5′-CTAGTCTAGAATATTGCATGAGTAACTGCTGGT-3′) The PCR product was cloned into pmirGLO dual-Luciferase vector (E1980, Promega, Madison WI USA) with *Xho* I and *Xba* I (R0145, *NEW ENGLAND* BioLabs, Ipswich MA USA) restriction enzyme sites. miR-18b-5p (forward primer, 5′- CGCGGATCCACCATGGTGATTTAATCAGA-3′ and reverse primer, 5′- CCGCTCGAGCCGTTCAAATCATTTCTCAA-3′) and miR-206 (forward primer, 5′-CGCGGATCCATTCTTCACACTTCTCACTT-3′ and reverse primer, 5′-CCGCTCGAG ACGAAGAAGTCAACAGCATA-3′) were amplified from NSC-34 cDNA by PCR. The PCR product was cloned into pCDNA3 vector (V79020, Invitrogen, Carlsbad CA USA) with *BamH I* and *Xho* I (R0136,R0146,*NEW ENGLAND* BioLabs, Ipswich MA USA) restriction enzyme sites. The mouse Mctp1 was amplified by PCR from NSC-34 cDNA (forward primer, 5′-CCCAAGCTTATGTACCAGTTGGATATCACACTA-3′ and reverse primer, 5′-CCCAAGCTTGCCAAGGTTGTTTTTTCTTCC-3′). The PCR product was cloned into mCherry C1 (632524, Clontech, Mountain View CA USA) with *Hind* III (R0104, *NEW ENGLAND* BioLabs, Ipswich MA USA) restriction enzyme sites.

The mouse Rarb was amplified by PCR from NSC-34 cDNA (forward primer, 5′-CCGCTAGCATGAGCACCAGCAGCCACGC-3′ and reverse primer, 5′-CCACCGGTCTGCAGCAGTGGTGACTGAC-3′) Table S1. The PCR product was cloned into eGFP N1 (PT3027–5, Clontech, Mountain View CA USA) with *Nhe* I and *Age* I (R0131, R0552, *NEW ENGLAND* BioLabs, Ipswich MA USA) restriction enzyme sites. The 3’UTR of Mctp1 and Rarb mutagenesis was performed by KOD-Plus-Mutagenesis Kit (F0936K, TOYOBO, Osaka Japan). Primer sequences are given in Table S1.

### Luciferase assay

The 3’UTR of Mctp1 and Rarb analysis was performed using (pmirGLO dual-luciferase vector (E1330, Promega, Madison WI USA)). pmirGLO-Mctp1 and Rarb reporter were transiently transfected in NSC-34 mouse motor-neuron-like cells (contNSC-34) with miR-206. The 3’UTR of HIF1α analysis was performed using (pmirGLO dual-luciferase vector (Promega)). pmirGLO- HIF1α reporter were transiently transfected in contNSC-34 cells with miR-18b-5p. The luciferase activity was measured 48 h after the transfection and normalized using Dual-luciferase Reporter System (E1980, Promega, Madison WI USA) according to the manufacturer’s instruction.

### NSC-34 cell lines culture, cell differentiation with retinoic acid and immunofluorescence

NSC-34 mouse motor neuron-like cell lines (contNSC-34, wtNSC-34 (human SOD1) and mtNSC-34 (human SOD1 (G93A)) kindly provided by H Ryu, Korea Institute of Science and Technology, Seoul, Korea) were grown in Dulbecco’s modified Eagle’s medium (SH30243, Hyclone, Logan UT USA) supplemented with 10% FBS (16,000,044, Gibco, Grand Island NY USA),100 U/ml penicillin, 100 μg/ml streptomycin (15140–122, GIBCO Grand Island NY USA). NSC-34 cells were differentiated in DMEM with 1% FBS, 100 U/ml penicillin, 100 μg/ml streptomycin and 20 μM all-trans-RA (R2625, Sigma, Burlington MA USA). Cells were fixed at room temperature using 4% paraformaldehyde washed with PBS. Non-specific proteins were blocked by incubation in PBS containing 0.05% Bovine Serum Albumin (82–100-6, Millipore, Kankakee illimois USA) and 0.03% Triton X-100 (T8787, SIGMA, St. Louis MO USA) and treated with primary antibodies were anti-Oct4 (ab27985, abcam, Cambridge, CB2 0AX, UK), anti-Nanog (ab80892, abcam, Cambridge, CB2 0AX, UK), anti-Nestin (ab22035, abcam, Cambridge, CB2 0AX, UK), anti-Sox2 (ab97959, abcam, Cambridge, CB2 0AX, UK), anti-choline acetyltransferase (AB144P, Chemicon), HLXB9 polyclonal antibody (PA5–23407, Thermo Fisher, Rockford IL USA), MAP2 (Santa Cruz, Dallas Texas USA), anti-SOD1 (abcam, Cambridge, CB2 0AX, UK), Proteostat Aggresome Detection kit (Enz-51,035-k100, Enzo Life Science, Farmingdale NY USA). Cells were then incubated with fluorescence-labeled secondary antibodies, which are Alexa Fluor 488, 555 and 594 (Life Technologies) and finally mounted on micro slides by using Aqueous/Dry Mounting Medium (MO1, biomeda, Foster City CA) with DAPI (D1306, Thermo, Eugene Oregon USA). Imaging was performed using a confocal microscope (LEICA STED CW). To measure MAP2 staining neurites, at least 30 neurons were analyzed from three different experiments. 20x magnification images were acquired. ImageJ software was used to determine the average neurite length.

### Subcellular fractionation

wt and mtNSC-34 cells were grown in a 10 cm dish and they were harvested in 450 ul of ice-cold buffer A (10 mM HEPES at pH 7.9, 10 mM KCl, 1 mM dithiothreitol [DTT], and 0.1 mM EDTA at pH 8.0). NSC-34 WT and MT cells dispersed by pipetting and incubated for 25 min on ice. Then 5 μl of 10% NP-40 was added, and cells were incubated for 2 min on ice. The nuclei were precipitated by centrifugation at 5000 rpm for 3 min at 4 °C. The supernatant was taken as the cytoplsamic fraction.

### RNA-seq

Three sets of wt and mtNSC-34 cells were grown and harvested with each set from a separate passage of single cell line. Following subcellular fractionation, transcriptomes of 12 samples were analyzed by RNA-seq (Macrogen Inc.), the Illumina standard kit was used according to the manufacturer’s protocol. Briefly, 3 μg of each total RNA sample was used for polyA mRNA selection using streptavidin-coated magnetic beads, followed by thermal mRNA fragmentation. The fragmented mRNA was subjected to cDNA synthesis using reverse transcriptase (SuperScript II) and random primers. The cDNA was further converted into double stranded cDNA and, after an end repair process (Klenow fragment, T4 polynucleotide kinase and T4polymerase), was finally ligated to Illumina paired end (PE) adaptors. Size selection was performed using a 2% agarose gel, generating cDNA libraries ranging in size from 200 to 250 bp. Finally, the libraries were enriched using 10 cycles of PCR and purified by the QIAquick PCR purification kit (28,106, Qiagen, PL Venlo Netherlands). The enriched libraries were diluted with Elution Buffer to a final concentration of 10 nM. Each library was run at a concentration of 8 pM on one Genome Analyzer (GAIIx) lane using 53 bp sequencing. Reads were then processed and aligned to the mouse genome UCSC build mm9 using GSNAP. The unit of measurement is Reads Per Kilobase of exon per Million fragments mapped (RPKM).

### Differential expression analysis

The expressed transcripts was quantified using Kallisto. DESeq2 and edgeR were used to identify transcripts that were differentially expressed between wt and mtNSC-34 cells. Different expression level of each transcript was defined as those that satisfied 2 criteria: |log_2_(fold-change)| > 1 and *p* < 0.01 after the Benjamini-Hochberg correction in DEseq2 and edgeR.

### Reverse transcription quantitative PCR (RT-qPCR)

Total RNA was extracted from wt and mtNSC-34 cells by TRIzol reagent (5741, MRC, Cincinnati OH USA). RNA was measured in a spectrophotometer at 260-nm absorbance. RNA analysis was conducted as follows. Fifty nanograms of RNA were used as a template for quantitative RT-PCR amplification, using SYBR Green Real-time PCR Master Mix (QPK201, Toyobo, Osaka Japan). Primers were standardized in the linear range of cycle before the onset of the plateau. Primer sequences are given in supplementary table 3 and 4. Mouse and human GAPDH was used as an internal control. Two-step PCR thermal cycling for DNA amplification and real-time data acquisition were performed with an ABI StepOnePlus™ Real-Time PCR System using the following cycle conditions: 95 °C for 1 min × 1 cycle, and 95 °C for 15 s, followed by 62 °C for 1 min × 50 cycles. Fluorescence data were analyzed by the ABI StepOnePlus software and expressed as C_t_ the number of cycles needed to generate a fluorescent signal above a predefined threshold. The ABI StepOnePlus software set baseline and threshold values. Expression of each gene was normalized to Gapdh and expression of each miRNA was normalized to U6. The fold change in mRNA and miRNAs expression versus controls calculated using the 2^(−ΔΔCT)^ method. For miRNAs RT-qPCR, 50 ng of total RNA was reverse transcribed, using GenoExplore microRNA RT-qPCR Kit (2001, Geno Sensor Corporation, Tempe, Arizona 85,282 USA), and subsequently quantified using specific primers (GenoExplorer microRNA RT-qPCR primer sets (2003, Geno Sensor Corporation, Tempe, Arizona 85,282 USA)) for U6, miR-18b-5p and miR-206 (mouse and human).

### Human spinal cord samples

Post mortem spinal cord specimens from six normal controls and one with fALS (G86S) were used (supplementary Table S6). Control spinal cord samples were obtained from The Netherlands Brain Bank and the guidelines by The Netherlands Brain Bank were followed. Post mortem spinal cord specimens from fALS (G86S) spinal cord and blood samples from fALS (G17S) were analyzed with Institutional permission under Review Board in Seoul National University Hospital.

### Generation of iPS cell lines

Peripheral blood mononuclear cells (PBMC) isolated from whole blood using the Ficoll-Paque (17–1440-03, GE Healthcare Life Sciences, Marlborough MA USA) were cultured and expanded in StemPro-34 medium(10,639,011, GIBCO, Grand Island NY USA) supplemented with 1% penicillin-streptomycin (15,140,122, Life technologies, Grand Island NY USA), hSCF 100 ng/mL, hFLT-3100 ng/mL, hIL-3 20 ng/mL, and hIL-6 20 ng/mL (all of them from Peprotech, Rocky Hill, NJ USA). 1 × 106 PBMC were transduced overnight with Sendai viruses containing Oct3/4, Sox2, Klf4, and cMyc (A16517, CytoTune®-iPS Sendai Reprogramming Kit, Life technologies, Grand Island NY USA) at multiplicity of infection (MOI) of 5. After 3 days, the transduced cells were plated on the 20μg/ml mitomycin C (M4287, SIGMA, St. Louis MO USA) treated-Human scrotum foreskin fibroblasts (HFFs) in Cell Start-coated 35 mm dishes in complete StemPro-34 medium without cytokines and were changed medium every day until iPSCs started transitioning. And then, colony-formed iPSCs were replaced the mixture of the StemPro-34 medium without cytokines and DMEM F/12 with 15% Knockout SR, 40 ng/ml bFGF, 1% nonessential amino acids, 50 U/ml of penicillin, 50 μg/ml streptomycin and 0.1 mM 2-mercaptoethanol (all of them from GIBCO, Grand Island NY USA). At day 30 or later, colonies were mechanically picked and passaged onto freshly mitotically inactivated HFFs. The iPSC colonies were picked for expension.

### Generation of neural stem cell from iPSC

Colonies were detached by 2 mg/ml dispase and transferred in embryoid body (EB) medium that contains Essential 6 Medium supplemented with 15% knockout SR (10828–028, Gibco, Grand Island NY USA), 50 U/ml of penicillin, 50 μg/ml streptomycin (15140–122, GIBCO Grand Island NY USA) to 60-mm incoated bacterial plate at 37 for 5-7 days with change of medium every single day. And then, formed EBs transferred to Cell Start coated-35-mm culture dish. The culture method has been used previously [43]. When EBs attached to dish after 2–3 days, changed 0.5% N2 in DMEM/F12 supplemented with 1% nonessential amino acids, 50 U/ml of penicillin, 50μg/ml streptomycin and 0.1 mM 2-mercaptoethanol to the same based-medium plus 1% N2 supplement and 40 ng/ml b-fibroblast growth factor every other day until neural structures appeared. These neural structures were mechanically isolated and cultured floating in the medium and then became spheres. These spheres were fragmented mechanically and cultured onto Cell start-coated culture dished for 1 day and treated accutase (A11105–01, Gibco, Grand Island NY USA) at 37 °C incubator or 1 h. Neural stem cells were cultured in DMEM/F12 supplemented with 1% nonessential amino acids, 50 U/ml of penicillin, 50 μg/ml streptomycin and 0.1 mM 2-mercaptoethanol plus 0.5% N2 supplement and 40 ng/ml b-fibroblast growth factor onto the Cell Start-coated plates.

### Differentiation of neural stem cells into motor neurons

Neural stem cells were cultured in DMEM/F12 with 1% nonessential amino acids, 50 U/ml of penicillin, 50 μg/ml streptomycin and 0.1 mM 2-mercaptoethanol, 0.5% N2 and 40 ng/ml b-FGF for 2 days onto the Cell Start with 1 μg/ml laminin and 5μg/ml heparin-coated plates and then changed neural induction medium, which consists of the mixture of DMEM/F12 and neurobasal medium(A3582901, GIBCO, Grand Island NY USA) supplemented with 0.1 mM 2-mercaptoethanol, 0.5% N2 supplement (21985–023, 17,502–048, GIBCO, Grand Island NY USA) and 40 ng/ml b-fibroblast growth factor), 10 ng/ml neural growth factor (13,257,019, Invitrogen, Grand Island NY USA), 10 ng/ml sonic hedgehog (8908-SH, R&D Systems, Minneapolis MN USA), 10 μM forskolin and 1 μM retinoic acid (R2625, Sigma, Burlington MA USA), glial cell-derived neurotrophic factor(GDNF; 212-GD), brain-derived neurotrophic factor (BDNF; 248-BDB), ciliary neurotrophic factor, insulin-like growth factor 1 and neurotrophin-3 (NT3) (267-N3, R&D Systems, Minneapolis MN USA), all at 10 ng/ml, every day or every other day for a week [44].

### Confocal microscopy

Immunofluorescence staining and confocal microscopy was used to determine mouse anti-Chat (AB144P, Chemicon, Tumecula CA USA). Images were analyzed using a spinning disk confocal microscope (Leica, Buffalo Grove IL USA). Deconvolution and 3-dimensional construction of the confocal image was performed by AQI-X-COMBO-CWF program (Media cybernetics Inc., Rockville, MD, USA). Control experiments were performed in the absence of primary antibody or in the presence of blocking peptide.

### Statistical analysis

The data are presented as the mean ± standard error of the mean (SEM). Data analysis was performed by Student’s *t* test or one-way ANOVAs followed by Mann-Whitney and Kruskal-Wallis tests. Differences were considered statistically significant when *p* < 0.05.

## Results

### SOD1 mutation (G93A) induces apoptosis by aberrant gene expression

**Fig. 1:**
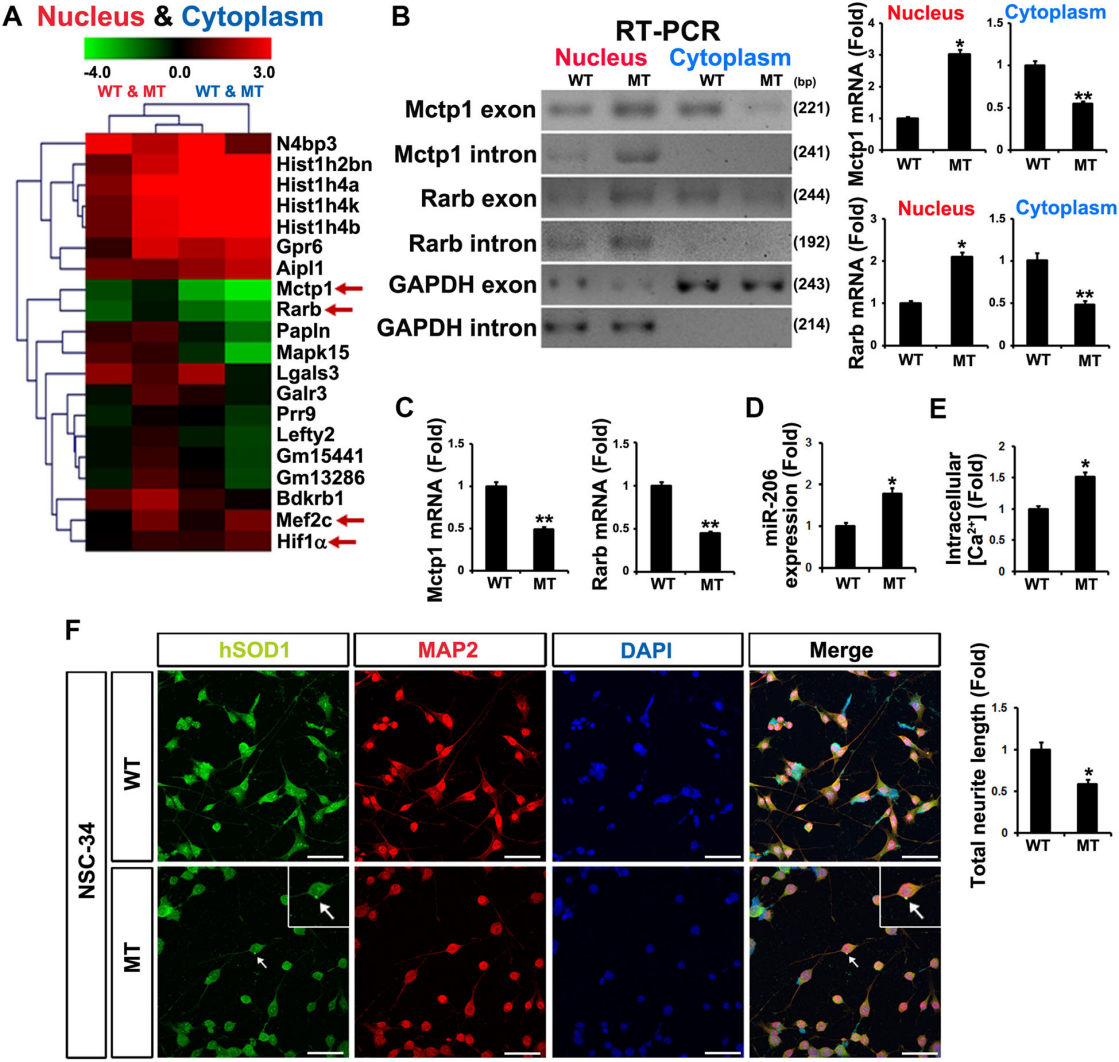
SOD1 mutation (G93A) is related to altered gene expression and induces apoptosis. **a** The heat map of RNA-seq from subcellular fractionized wt and mtNSC-34 stable cells showed that relative expressions of Hif1α, Mef2c, Mctp1 and Rarb are altered by SOD1(G93A). The relative expression genes was displayed as colors: higher (red) or lower (green). The heat map represents the average of three samples. **b** RT-PCR analysis explained that Mctp1 and Rarb introns are upregulated in nucleus of mtNSC-34 cells and Mctp1 and Rarb exons are highly downregulated in cytoplasm of mtNSC-34 cells. **c** RT-qPCR analysis showed that Mctp1 and Rarb transcripts are decreased in mtNSC-34 cells. **d** miR-206 expression was induced in mtNSC-34 cells. **e** and **f** SOD1 aggregation (arrow) prevents neuronal differentiation (MAP2) and increases intracellular Ca^2+^ levels (WT (0.072) versus MT (0.108) in fluorescence intensities from baseline 490/525 ratio) in mtNSC-34 cells. Scale bar, 40 μm. Significantly different at *, *p* < 0.05; **, *p* < 0.005. The experiments were replicated 3 times

**Fig. 2:**
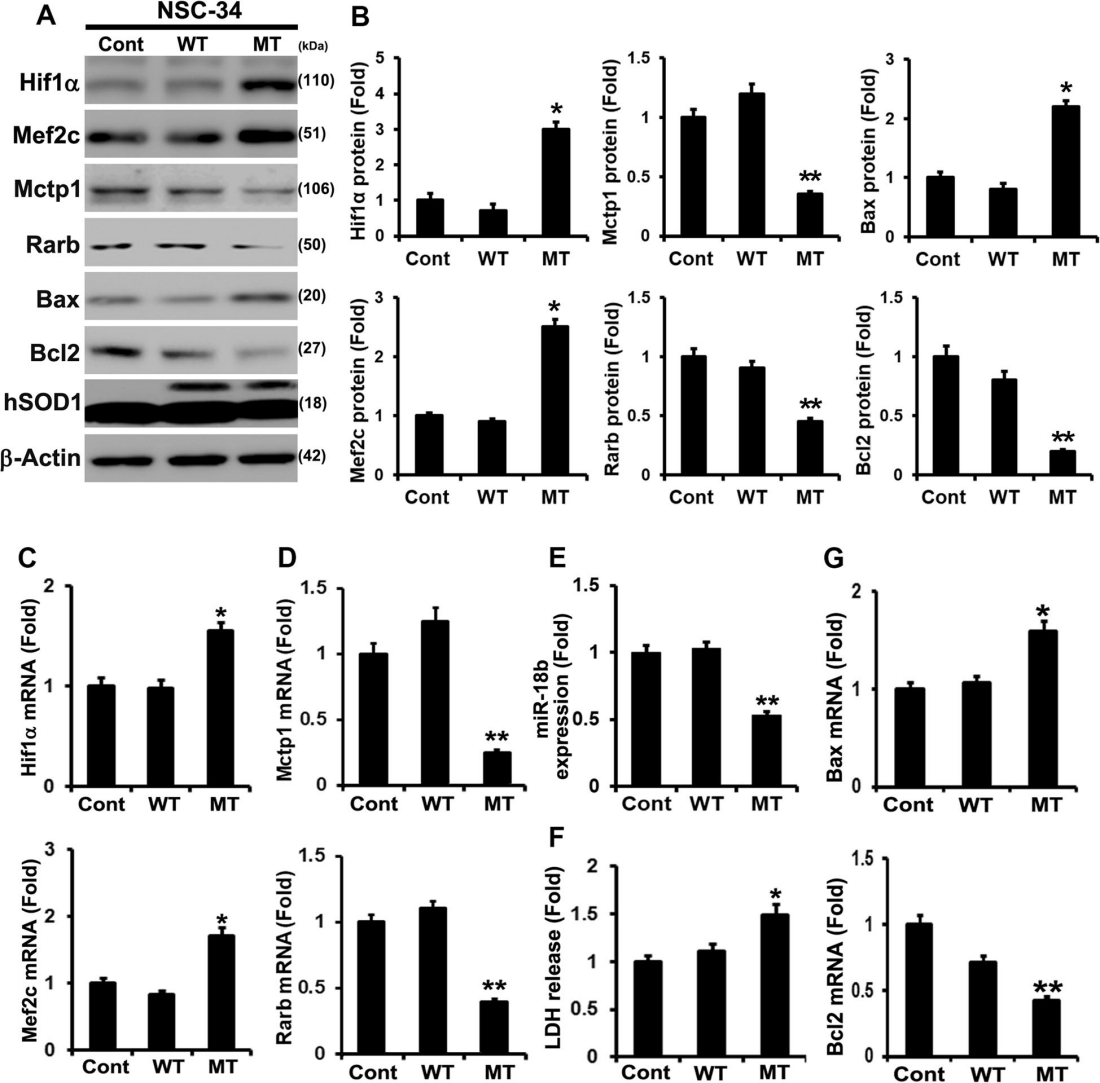
SOD1 mutation (G93A) induces apoptosis. **a** Western blot analysis verified the protein levels of Hif1α, Mef2c, Mctp1, Rarb, Bax, and Bcl2 in three different NSC-34 cell lines. **b** Quantification analysis of western blot. The data represent the average ± SEM of 3 separate experiments. Significantly different at *, *p* < 0.5; **, *p* < 0.05. **c** Hif1α and Mef2c transcripts were increased in mtNSC-34 cells. **d** Mctp1 and Rarb transcripts were decreased in mtNSC-34 cells **e** miR-18b (miR-18b-5p) was reduced in mtNSC-34 cells. **f** LDH releases analysis explained that SOD1 mutation (G93A) induces apoptosis. **g** Bax mRNAs were upregulated and Bcl2 mRNAs were downregulated in mtNSC-34 cells. Significantly different at *, *p* < 0.05; **, *p* < 0.005. The experiments were replicated 3 times

To study the mechanism of RNA biogenesis by SOD1 mutation, we performed transcriptome analysis to identify RNA processing variation via subcellular fractionized RNAs in NSC-34 hSOD1 (wtNSC-34) and hSOD1 (G93A) (mtNSC-34) stable cell lines (Fig. 1a). By performing comparative RNA-seq analysis between the nucleus and cytoplasm of WT and mtNSC-34 cells, we identified significant changes in Mctp1 and Rarb expression (Fig. 1a). The heat map shows that Mctp1 and Rarb were upregulated in the nucleus, but greatly downregulated in the cytoplasm of mtNSC-34 cells (Fig. 1a). To validate Mctp1and Rarb transcripts, we carried out reverse transcriptase PCR (RT-PCR) analysis and confirmed the presence of Mctp1 and Rarb mRNAs (Fig. 1b). To understand how Mctp1 and Rarb transcripts are regulated in mtNSC-34 cells, we performed revers transcription quantitative PCR (RT-qPCR) analysis in WT and mtNSC-34 cells (Fig. 1c). We found that Mctp1 and Rarb mRNAs were low in mtNSC-34 cells (Fig. 1c). These results led us to assume that Mctp1 and Rarb transcripts might be post-transcriptionally regulated or deficiently transported in the cytoplasm of mtNSC-34 cells. For the reason that miRNAs are one of the most representative the post-transcriptional regulators [45–47], we focused on the post-transcriptional regulation and identified that Mctp1 and Rarb mRNAs are targeted by miR-206 (Additional file 1: Fig. S1A). Furthermore, we discovered that miR-206 was remarkably upregulated in mtNSC-34 cells (Fig. 1d). Moreover, we investigated whether or not Mctp1 and Rarb were related to calcium signaling and neuronal differentiation, respectively, and measured intracellular Ca^2+^ levels and total neurite length in WT and mtNSC-34 cells (Fig. 1e and f). As expected, the intracellular Ca^2+^ levels increased, and the neurite outgrowth decreased significantly in mtNSC-34 cells with the aggregation of SOD1 (G93A) (Fig. 1e and f). These results showed that the downregulated Mctp1 and Rarb could stimulate the alteration of calcium signaling and cell differentiation in mtNSC-34 cells, respectively. From the RNA-seq results, we also identified that Hif1α and Mef2c, which are regulated by Hif1α [48], increased in the nucleus and cytoplasm of mtNSC-34 cells (Fig. 1a). Interestingly, Mef2c has been reported to regulate miR-206 as a transcription factor [49]. We validated that Hif1α and Mef2c expression was significantly elevated in mtNSC-34 cells (Fig. 2a and b). From these results, we also hypothesized that an unknown target miRNA of Hif1α might be downregulated, and that the upregulated Hif1α could upregulate Mef2c expression serially. We also identified that the miR-18b-5p was target of Hif1α mRNAs (Additional file 1: Fig. S1B) [50]. RT-qPCR analysis confirmed that miR-18b-5p was significantly reduced in mtNSC-34 cells (Fig. 2e). We also confirmed that only mtSOD1 (G93A) were associated with miR-18b-5p, Hif1α, Mef2c, Mctp1 and Rarb in NSC-34 (control, wtSOD1, and mtSOD1) stable cell lines (Fig. 2a-e). Owing to the fact that SOD1 mutations cause apoptosis [51], we measured the levels of Bax and Bcl2 as pro- and anti-apoptotic markers. Bax expression was increased, while Bcl2 expression was decreased in mtNSC-34 cells (Fig. 2a, b, and g). We then measured the amount of lactate dehydrogenase (LDH) released to observe apoptosis in NSC-34 cell lines. As expected, LDH release increased in mtNSC-34 cells (Fig. 2f). These results suggested that downregulated miR-18b-5p might sequentially control Hif1α, Mef2c, miR-206, Mctp1, and Rarb expression in SOD1 mutation.

### Downregulated miR-18b-5p directly upregulates Hif1α and enhances apoptosis in mtNSC-34 cells

**Fig. 3:**
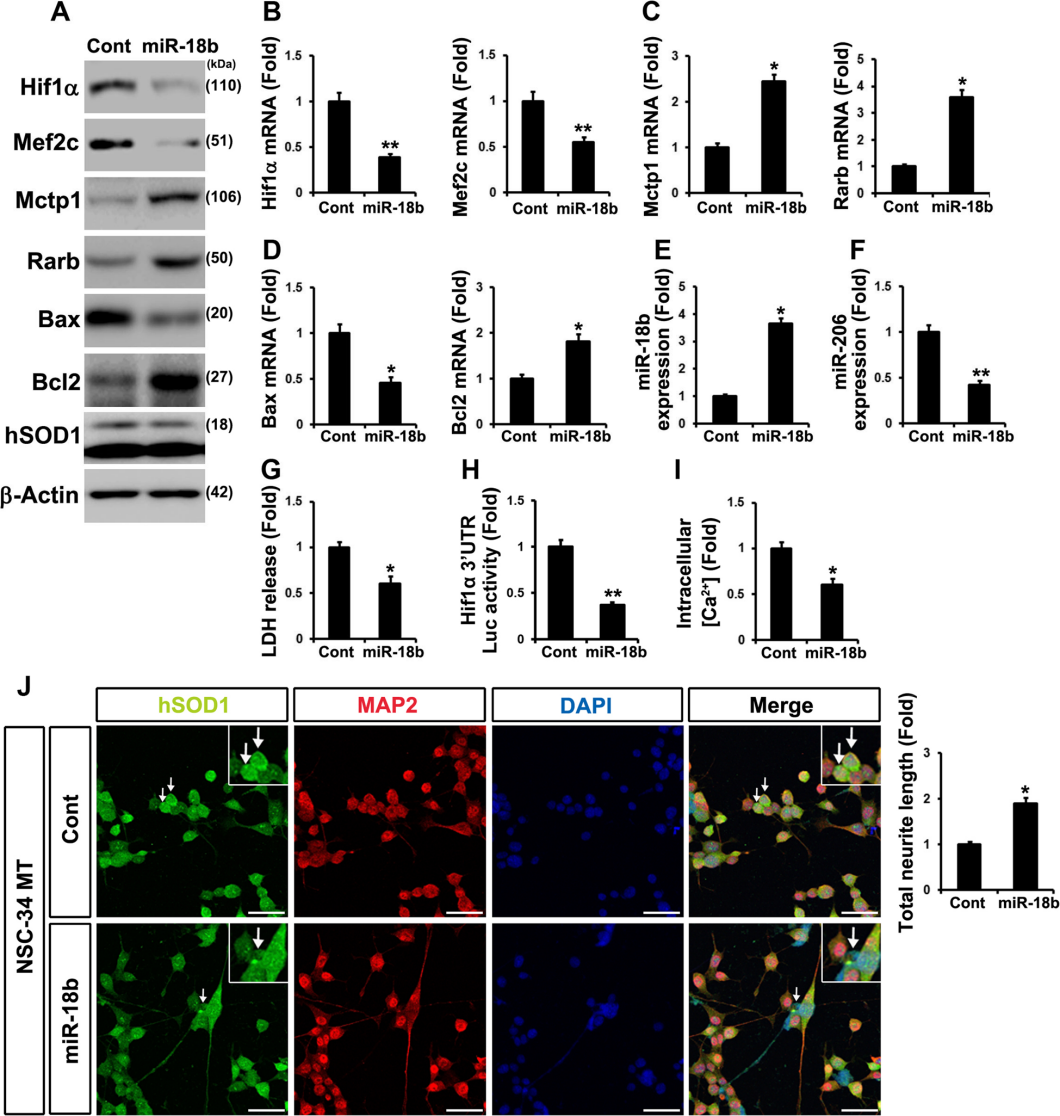
miR-18b (miR-18b-5p) regulates Hif1α and reduces apoptosis in mtNSC-34 cells. **a** Overexpressed miR-18b (miR-18b-5p) decreased Hif1α and Mef2c proteins. Both Mctp1 and Rarb expression were increased by miR-18b (miR-18b-5p). Downregulated Bax and upregulated Bcl2 by miR-18b (miR-18b-5p) diminished apoptosis in mtNSC-34 cells. **b** RT-qPCR analysis showed low expression of Hif1α and Mef2c mRNAs. **c** Mctp1 and Rarb transcripts were highly expressed by miR-18b (miR-18b-5p). **d** Bax mRNAs were decreased and Bcl2 mRNAs were increased by overexpressed miR-18b. **e** miR-18b (miR-18b-5p) was overexpressed in mtNSC-34 cells. **f** miR-206 was reduced by miR-18b (miR-18b-5p). **g** LDH release analysis explained that transfected miR-18b (miR-18b-5p) restores apoptosis. **h** Luciferases assay with 3′ UTR of Hif1α showed that Hif1α is target of miR-18b in contNSC-34 cells. **i** and **j** Overexpression of miR-18b (miR-18b-5p) enhanced neuronal differentiation (MAP2) and attenuated intracellular Ca^2+^ levels (Cont (0.098) versus miR-18b (miR-18b-5p) (0.051) in fluorescence intensities from baseline 490/525 ratio) in mtNSC-34 cells. Empty vector served as a negative control (Cont). Arrow represents SOD1 aggregation (green). Scale bar, 40 μm. Significantly different at *, *p* < 0.05; **, *p* < 0.005. The experiments were replicated 5 times

In order to examine whether or not sequential events of downregulated miR-18b-5p are associated with apoptosis, we used RNAi method to reduce miR-18b-5p in contNSC-34 cells. Transfected Anti-18b (anti-miR-18b-5p) elevated Hif1α and Mef2c proteins, while it decreased Mctp1 and Rarb proteins (Additional file 2: Fig. S2A). RT-qPCR analysis also confirmed that mRNAs of Hif1α and Mef2c were decreased, and those of Mctp1 and Rarb were increased (Additional file 2: Fig. S2B-E). Downregulated miR-18b-5p was found to finally induce apoptosis, because expression of Bax increased, while expression of Bcl2 decreased. In addition, LDH release was also increased by anti-18b (anti-miR-18b-5p) (Additional file 2: Fig. S2A, F, G, and H). Interestingly, RT-qPCR analysis results showed that miR-206 was rapidly induced by anti-18b (anti-miR-18b-5p) (Additional file 2: Fig. S2I and J). To identify apoptosis by downregulated miR-18b-5p, we isolated mouse neural stem cells (mNSCs) from the brains of mice and transfected anti-18b (anti-miR-18b-5p). Flow cytometry analysis showed that anti-18b (anti-miR-18b-5p) elevates apoptosis in mNSCs (Additional file 2: Fig. S2K). Based on these results, we naturally wondered whether transfected miR-18b-5p could restored apoptosis. We ectopically expressed miR-18b-5p and measured the protein of Hif1α, Mef2c, Mctp1, Rarb, Bax, and Bcl2 in mtNSC-34 cells. Hif1α and Mef2c expressions decreased (Fig. 3a and b) while Mctp1 and Rarb expressions increased by increased miR-18b-5p (Fig. 3a and c). Ectopically overexpressed miR-18b-5p induced the reversible changes in Bax and Bcl2 expressions in mtNSC-34 cells (Fig. 3a and d). miR-206 was also downregulated by overexpressed miR-18b-5p (Fig. 3e and f). The LDH release assay showed that induced miR-18b-5p prevented apoptosis (Fig. 3g). We also confirmed that Hif1α was target of miR-18b-5p in contNSC-34 cells (Fig. 3h). In order to confirm that the overexpression of miR-18b-5p was mediated by the regulation of Ca^2+^ signaling and cell differentiation, we measured intracellular Ca^2+^ levels and total neurite length using the cell differentiation assay. We observed aggregated SOD1 in mtNSC-34 cells. Nevertheless, overexpressed miR-18b-5p cells not only reduced intracellular Ca^2+^ levels, but also increased neurite outgrowth (Fig. 3i and j). These results proved that miR-18b-5p was related to Hif1α expression and apoptosis.

### Hif1α directly regulates Mef2c expression and apoptosis

As we had mentioned previously in this article that decreased miR-18b-5p upregulated Hif1α, which subsequently increased Mef2c, we confirm that Hif1α regulated Mef2c. We applied RNAi to decrease Hif1α, and observed the regulation of Mef2c, miR-206, Mctp1, and Rarb in mtNSC-34 cells (Additional file 3: Fig. S3). The expression of Mef2c was reduced by siHif1α in mtNSC-34 cells (Additional file 3: Fig. S3A-C). Mctp1 and Rarb expressions were also increased (Additional file 3: Fig. S3A, D, and E). To prove that Hif1α restored apoptosis, we observed the downregulation of Bax and upregulation of Bcl2 by siHif1α in mtNSC-34 cells (Additional file 3: Fig. S3A, F, and G). We also indirectly identified that Mef2c, inhibited by siHif1α, decreased miR-206 (Additional file 3: Fig. S3H). In order to observe the reduced apoptosis, we applied the LDH assay and, as we expected, LDH release was reduced by siHif1α in mtNSC-34 cells (Additional file 3: Fig. S3I). These results suggested that Hif1α directly regulated Mef2c and contributed to apoptosis.

### miR-206 not only controls post-transcriptional regulation of both Mctp1 and Rarb, but also induces apoptosis

**Fig. 4:**
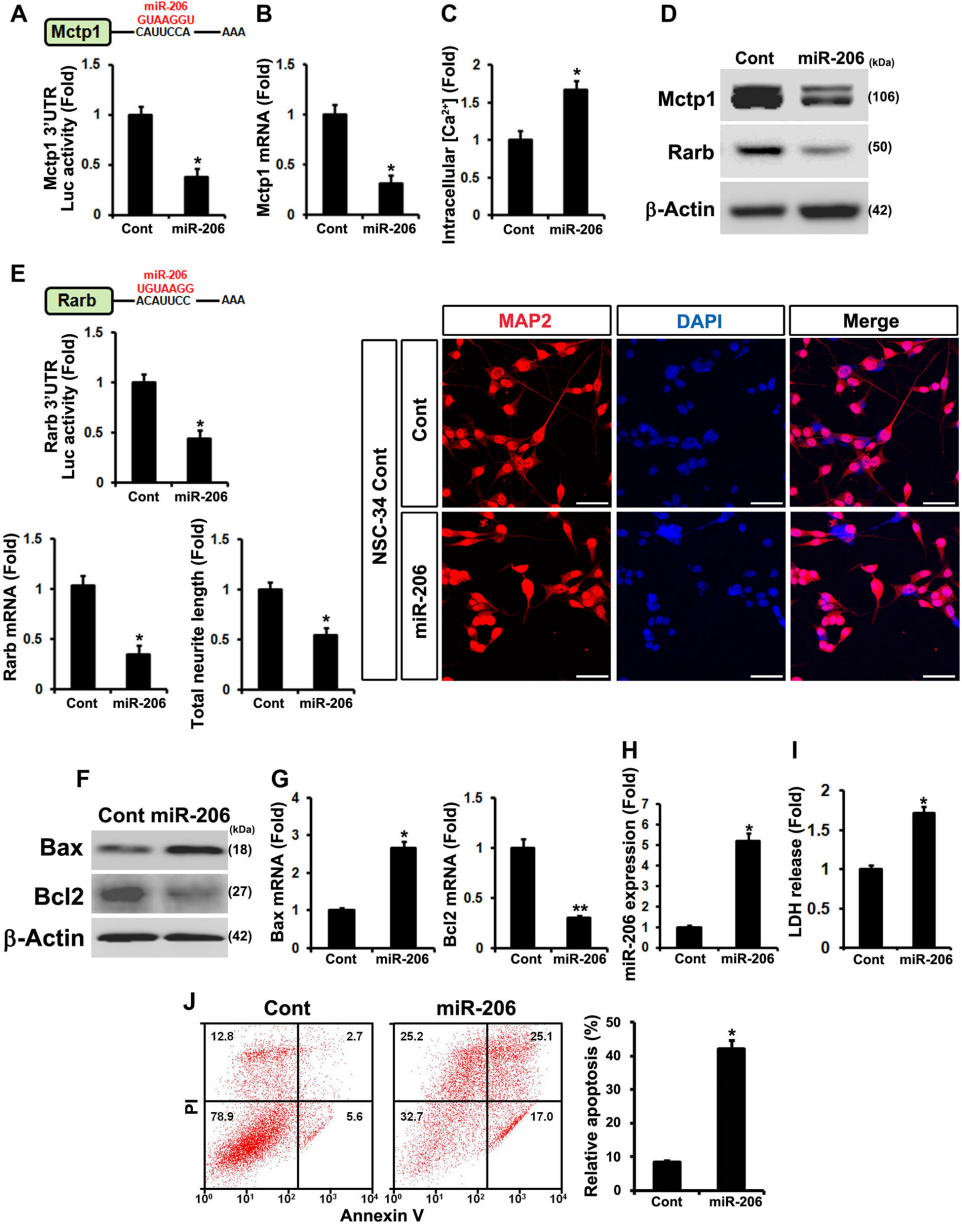
miR-206 post-transcriptionally regulates Mctp1 and Rarb and elevates apoptotic cell death. **a** and **b** Luciferases assay with 3′ UTR of Mctp1 showed that Mctp1 is target of miR-206. Mctp1 transcripts was downregulated by increased miR-206. **c** Intracellular Ca^2+^ levels (Cont (0.069) versus miR-206 (0.122) in fluorescence intensities from baseline 490/525 ratio) was enhanced by miR-206. **d** miR-206 controlled Mctp1 and Rarb protein expression. **e** Luciferase assay with 3′ UTR of Rarb also verified that Rarb is target of miR-206. Rarb mRNAs was decreased by miR-206. Neuronal cell differentiation (MAP2) was reduced by miR-206. Scale bar, 40 μm. **f** and **g** Both Bax protein and mRNAs were increased by miR-206. Bcl2 proteins and mRNAs were decreased by miR-206. **h** miR-206 was overexpressed in contNSC-34 cells. **i**) LDH release assay showed that miR-206 enhances apoptosis. **j** Flow cytometry analysis explained that overexpressed miR-206 activates apoptotic cell death in mouse NSCs. Empty vector served as a negative control (Cont). Significantly different at *, *p* < 0.05; **, *p* < 0.005. The experiments were replicated 3 times

To further investigate the roles of miR-206, we carried out a luciferase reporter assay using the 3′ UTR of Mctp1 and Rarb. Mctp1 was significantly downregulated and intracellular Ca^2+^ levels were increased by overexpressed miR-206 (Fig. 4a-d). Rarb levels were also decreased by miR-206 (Fig. 4d and e). The post-transcriptionally downregulation of Rarb by miR-206 also caused the neuronal differentiation (Fig. 4e). We then observed that overexpressed miR-206 induced apoptosis, because Bax expression was upregulated, while Bcl2 expression was downregulated by miR-206 (Fig. 4f and g). Overexpression of miR-206 was confirmed by RT-qPCR analysis and miR-206 considerably induced LDH release (Fig. 4h and i). We opted to have it reconfirmed by performing flow cytometry analysis of mNSCs overexpressing miR-206 ectopically. As a result, apoptosis was markedly induced (Fig. 4j). To identify whether or not miR-206 simultaneously regulates Mctp1 and Rarb, we then performed anti-206 (anti-miR-206) experiments. Mctp1 and Rarb proteins were significantly increased by transfected anti-206 (anti-miR-206) in mtNSC-34 cells (Additional file 4: Fig. S4A and G). Anti-206 (anti-miR-206) also reduced Bax and induced Bcl2 levels in mtNSC-34 cells (Additional file 4: Fig. S4A, D, E, and G). Mctp1 and Rarb transcripts by anti-206 (anti-miR-206) showed the same results as western blot in the mtNSC-34 cells (Additional file 4: Fig. S4B and C). In addition, the LDH assay using anti-206 (anti-miR-206) in mtNSC-34 cells showed that anti-206 (anti-miR-206) restored cell death (Additional file 4: Fig. S4F). These findings suggested that miR-206 directly regulates Mctp1 and Rarb, and then induces apoptosis. To confirm that miR-206 post-transcriptionally regulates Mctp1 and Rarb, we deleted miR-206 binding sites from 3’UTR of Mctp1 and Rarb. We carried out a luciferase reporter assay using the mutation 3′ UTR of Mctp1 and Rarb. It did not show any significant change in miR-206 expression (Additional file 4: Fig. S4H and I).

### Post-transcriptionally downregulation of Mctp1 and Rarb inhibited calcium signaling and neuronal cell differentiation in mtNSC-34 cells

**Fig. 5:**
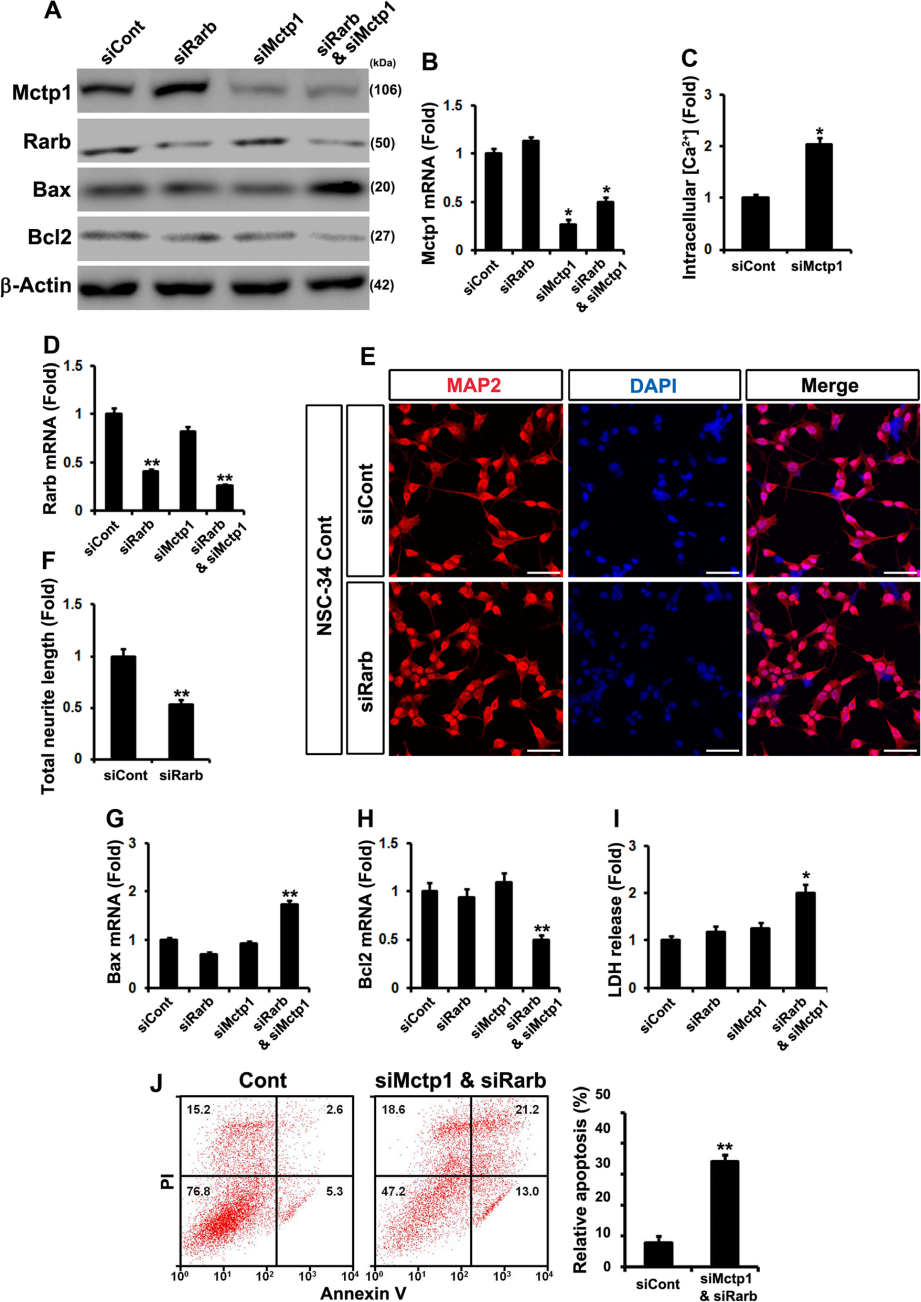
Downregulated Mctp1 and Rarb are associated with apoptotic cell death. **a** Bax proteins were increased by double knockdown of Mctp1 and Rarb. Bcl2 expression was decreased by siMctp1 and Rarb. **b** Mctp1 transcripts were downregulated. **c** Knockdown of Mctp1 enhanced Intracellular Ca^2+^ levels (siCont (0.063) versus siMctp1 (0.131) in fluorescence intensities from baseline 490/525 ratio). **d** Rarb transcripts were downregulated. **e-f** Transfected siRarb reduced neuronal cell differentiation (MAP2). Scale bar, 40 μm. **g** and **h** Double knockdown of Mctp1 and Rarb increased Bax transcripts and decreased Bcl2 transcripts. **i** Transfected siMctp1 and siRarb activated LDH release. **j** Flow cytometry verified that downregulated Mctp1 and Rarb significantly induce apoptotic cell death in mouse NSCs. Scrambled siRNA served as a negative control (siCont). Significantly different at *, *p* < 0.05; **, *p* < 0.005. The experiments were replicated 3 times

We have mentioned earlier in this article that downregulated miR-18b-5p regulated Hif1α which are related to multiple gene expression directly or indirectly (Mef2c, miR-206, Mctp1 and Rarb). These results indicated that Mctp1 and Rarb downregulated by miR-206 induced apoptosis in mtNSC-34 cells, and downregulation of Mctp1 and Rarb is related to the inhibition of calcium signaling and cell differentiation, respectively. To explore whether or not Mctp1 and Rarb deficiency directly induces apoptosis, we used RNAi methods in contNSC-34 cells. Interestingly, siMctp1 enhanced the intracellular Ca^2+^ concentration, but did not affect Bax and Bcl2 expression (Fig. 5a-c and g-h). siRarb inhibited cell differentiation but did not induce a significant change in Bax and Bcl2 expression (Fig. 5a and d-h). Due to the fact that miR-206 simultaneously decreased Mctp1 and Rarb, we inhibited Mctp1 and Rarb simultaneously. Bax expression was increased and Bxl2 was decreased by double-knockdown of Mctp1 and Rarb in contNSC-34 cells (Fig. 5a). RT-qPCR analysis also verified the upregulated Bax transcripts and downregulated Bcl2 transcripts by co-transfected siMctp1 and siRarb (Fig. 5g and h). LDH also was released more following the simultaneous reduction in Mctp1 and Rarb (Fig. 5i). Flow cytometry analysis also demonstrated enhanced apoptosis following ectopic downregulation of Mctp1 and Rarb in mNSCs (Fig. 5j). To investigate whether or not induced Mctp1 and Rarb had a direct therapeutic effect on apoptosis, we transfected Mctp1 and Rarb in mtNSC-34 cells and measured Bax and Bcl2 expression. The overexpressed Mctp1 and Rarb did not induce significant changes, as in the loss-of-function studies, but co-transfected Mctp1 and Rarb reduced Bax and induced Bcl2 in mtNSC-34 cells (Additional file 5: Fig. S5A-C). The LDH assay also showed that simultaneous overexpression of Mctp1 and Rarb reduced apoptosis in mtNSC-34 cells (Additional file 5: Fig. S5D). To study whether or not Mctp1 and Rarb significantly affected intracellular Ca^2+^ levels and neurite outgrowth respectively, we ectopically caused the occurrence of overexpression Mctp1 and Rarb. Mctp1 reduced intracellular Ca^2+^ levels, and Rarb enhanced neurite length in mtNSC-34 cells (Additional file 5: Fig. S5E, F, and G). Altogether, these gain- and loss-of-function studies concerning Mctp1 and Rarb imply that calcium signaling and neuronal cell differentiation are involved in important pathogenic mechanisms associated to SOD1 (G93A) mutations.

### Downregulation of the miR-18b-5p signaling pathway is involved in diverse SOD1 mutations and in vivo studies

**Fig. 6:**
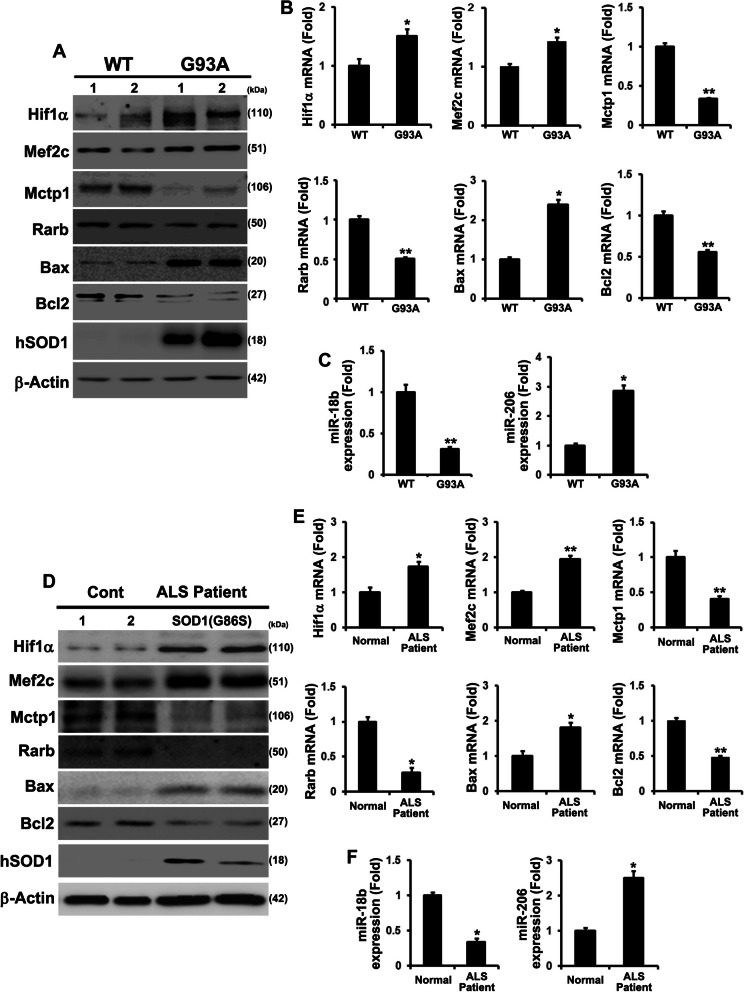
Downregulated miR-18b (miR-18b-5p) by SOD1 mutations contributes apoptotic cell death in SOD1(G93A) Tg mice and fALS patient spinal cord tissues. **a** Hif1α, Mef2c and Bax expression were increased in G93A mice. Mctp1, Rarb and Bcl2 proteins were decreased in G93A mice. **b** mRNAs of Hif1α, Mef2c and Bax was highly expressed in G93A mice. Mctp1, Rarb and Bcl2 transcripts were significantly downregulated in G93A mice. **c** miR-18b (miR-18b-5p) was deeply reduced and miR-206 was dramatically induced in G93A mice (*n* = 5). **d** The protein levels of Hif1α, Mef2c and Bax was upregulated in fALS (G86S) patient (Cervical and lumber). Mctp1, Rarb and Bcl2 proteins were decreased in fALS (G86S) patient. Normal spinal cord tissues (Cervical (control 1 and 2) served as a negative control (Cont). **e** The transcripts of Hif1α, Mef2c and Bax was highly upregulated in fALS (G86S) patient (Cervicals). Mctp1, Rarb and Bcl2 transcripts were significantly downregulated in fALS (G86S) patient. **f** miR-18b (miR-18b-5p) expression was importantly decreased and miR-206 was highly expressed in fALS (G86S) patient (Cervial and Lumber). Normal spinal cord tissues (Cervicals (control 1 and 2) served as a negative control (Cont). hSOD1 immunoreactivity on western blots of the insoluble fraction of the G93A mice and fALS (G86S) patients tissues. Significantly different at *, *p* < 0.05; **, *p* < 0.005

To identify the pivotal role of the miR-18b-5p pathway in other SOD1 mutations, we ectopically caused the overexpression of SOD1 (G85R and D90A) in contNSC-34 cells. Transfected G85R and D90A demonstrated that Hif1α and Mef2c expression was increased and Mctp1 and Rarb expression was decreased (Additional file 6: Fig. S6A-C). G85R and D90A also enhanced apoptosis by difference the expression of Bax and Bcl2 (Additional file 6: Fig. S6A and D). RT-qPCR analysis results showed that miR-18b-5p was downregulated and miR-206 was upregulated by G85R and D90A (Additional file 6: Fig. S6E-G). Furthermore, we sought to verify the miR-18b-5p pathway in G93A Tg and fALS (G86S) patient. We first compared the miR-18b-5p pathway in the spinal cords of WT and G93A Tg. The expression of Hif1α and Mef2c was highly increased, yet that of Mctp1 and Rarb was significantly decreased in G93A Tg (Fig. 6a and b). Increased Bax and decreased Bcl2 indicated the apoptosis occurring in G93A Tg (Fig. 6a and b). RT-qPCR results also showed that miR-18b-5p was downregulated and miR-206 was upregulated in G93A Tg (Fig. 6c). Further, we confirmed the miR-18b-5p pathway in the spinal cord of the G86S patient. Hif1α and Mef2c expression significantly increased, while the expression of Mctp1 and Rarb decreased in the G86S patient (Fig. 6d and e). The expression of Bax and Bcl2 was induced and reduced respectively in the G86S patient (Fig. 6d and e). RT-qPCR analysis showed decreased miR-18-5p and increased miR-206 in the G86S patient (Fig. 6f). These results strongly demonstrated that the miR-18b-5p pathway was generally involved in SOD1 mutation.

### Downregulated miR-18b-5p triggered apoptosis in differentiated MNs from hiPSC-derived NSCs of a SOD1 (G17S) fALS patient

**Fig. 7:**
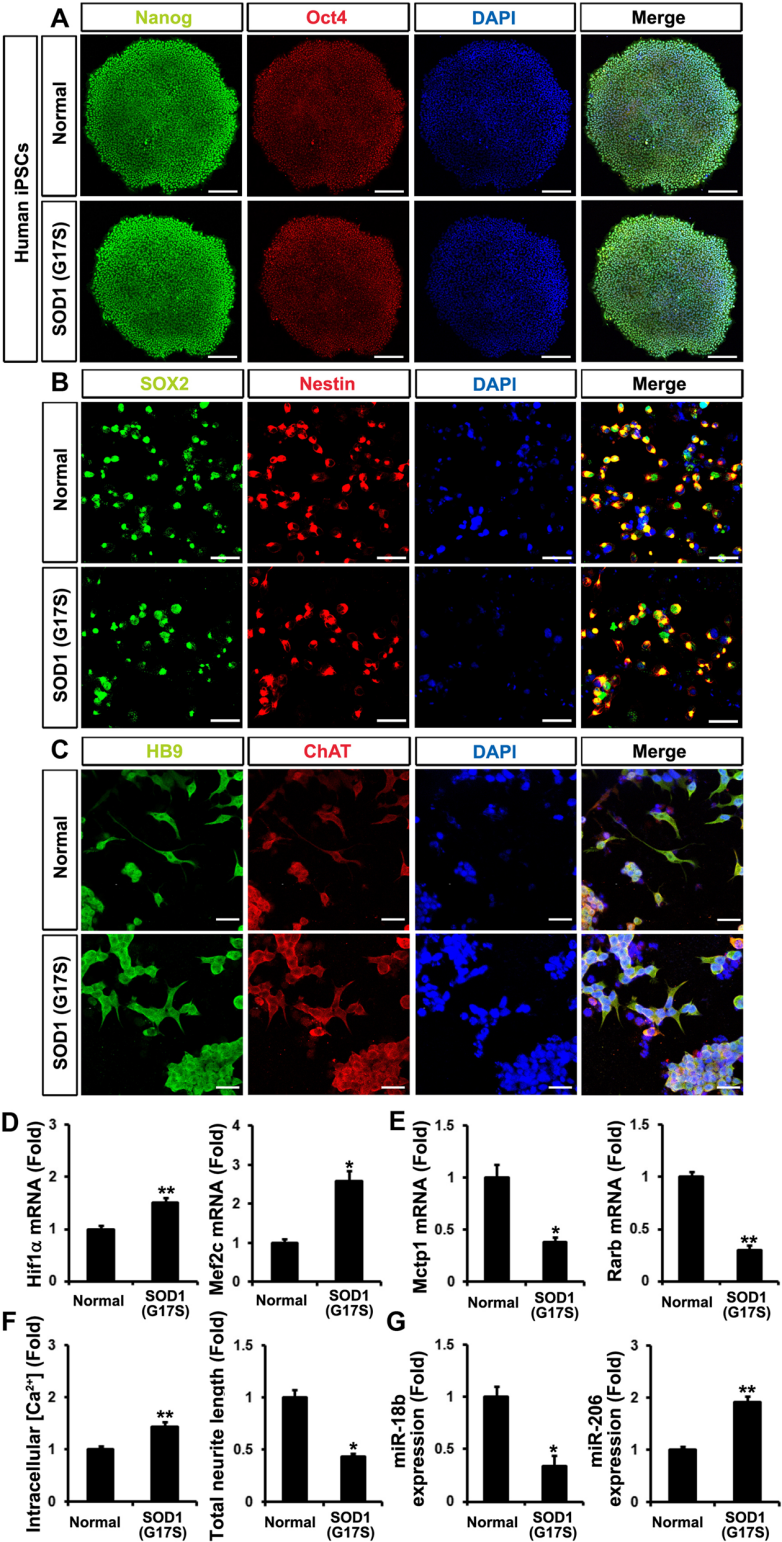
Downregulated miR-18b (miR-18b-5p) by SOD1 mutations is associated with apoptotic cell death in motor neuron from hiPSC-derived NSCs fALS SOD1 (G17S). **a** Nanog (green) and Oct4 (red) iPSC marker are expressed in hiPSCs (normal and fALS SOD1 (G17S). Scale bar, 20 μm. **b** Neural stem cells (SOX2 and Nestin) was generated from hiPSCs (normal and fALS SOD1 (G17S)). Scale bar, 40 μm. **c** Differentiated motor neurons from hNSCs were double-stained with HB9 and ChAT antibodies. Scale bar, 20 μm. **d** Hif1α and Mef2c transcripts were upregulated in differentiated motor neurons SOD1 (G17S). **e** The mRNAs levels of Mctp1 and Rarb was downregulated in differentiated motor neurons SOD1 (G17S). **f** Intracellular Ca^2+^ levels (Normal (0.149) versus SOD1 (G17S) (0.215) in fluorescence intensities from baseline 490/525 ratio) were enhanced in differentiated motor neurons SOD1 (G17S). Total neurite length was reduced in differentiated motor neurons SOD1 (G17S). **g** miR-18b (miR-18b-5p) expression was deeply low and mir-206 was highly expression in differentiated motor neurons SOD1 (G17S). Significantly different at *, *p* < 0.05; **, *p* < 0.005. The experiments were replicated 5 times

In the G86S patient study, we could not compare the normal lumber to G86S patient lumber tissues (Fig. 6d). However, we clearly confirmed miR-18b-5p pathway in cervical tissues. To support that miR-18b-5p pathway play a pivotal role in fALS patient motor neurons, we developed hiPSCs derived from WT and fALS SOD1 (G17S) patient blood. WT and G17S iPSCs were confirmed using pluripotency markers and RT-PCR (Fig. 7a and Additional file 7: Fig. S7A). We also generated NSCs, and the immunocytochemical staining demonstrated that SOX2 and Nestin expression were induced in WT and G17S NSCs (Fig. 7b). To validate the variation in miR-18b-5p, Hif1α, Mef2c, miR-206, Mctp1, and Rarb transcripts, we induced MNs from NSCs which were characterized by HB9 and ChAT (Fig. 7c). We confirmed that Hif1α and Mef2c transcripts significantly increased in G17S MNs (Fig. 7d). Mctp1 and Rarb mRNAs remarkably decreased in G17S MNs (Fig. 7e). We also measured intracellular Ca^2+^ levels and neurite length. As we expected, Ca^2+^ was highly accumulated and neuronal cell differentiation was inhibited in G17S MNs (Fig. 7f and Additional file 7: Fig. S7B). We also verified that Bax transcripts were upregulated and Bcl2 transcripts were downregulated in G17S MNs (Additional file 7: Fig. S7C). We also measured downregulated miR-18b-5p and upregulated miR-206 in G17S MNs (Fig. 7g). LDH release was increased in G17S MNs (Additional file 7: Fig. S7D). Altogether, these findings show that the downregulated miR-18b-5p systematically controls Hif1α, Mef2c, miR-206, Mctp1, and Rarb expression and finally induces apoptosis in ALS.

## Discussion

Recently, various pathological pathways and mechanisms have gradually been discovered for ALS and FTLD [13]. SOD1, FUS, and TDP-43 are representatively associated with ALS pathogenesis [2–9]. Specifically, RNA processing studies are based on the pathogenic mechanisms of FUS and TDP-43 because they have several functional and structural similarities [52, 53]. Although SOD1, which is the first discovered ALS-causing mutated gene and is linked only with the ALS phenotype, does not have much functional correlation with FUS and TDP-43, many researchers have attempted to study dysregulated RNA biogenesis with regard to SOD1 mutations [9, 13]. However, precise mechanisms of RNA metabolism in SOD1 mutations are still unclear. In the present study, we attempted to study abnormal RNA processing in SOD1 mutations in a different way compared to previous efforts. To uncover abnormal RNA expression in the nucleus and cytoplasm, we performed RNA-seq in nuclear and cytoplasmic subcellular fractions from wtNSC-34 and mtNSC-34 cells. Then, we identified that mutated SOD1 (G93A) up or downregulated several gene expression in the nucleus and cytoplasm. Among them, we discovered that Hif1α and Mef2c were notably upregulated in the nucleus and cytoplasm of mtNSC-34 cells, while Mctp1 and Rarb were upregulated in the nucleus, but were strongly downregulated in the cytoplasm. These results led us to hypothesize that the altered expression of Mctp1 and Rarb might be affected by post-transcriptional regulation. We identified that Mctp1 and Rarb are target genes of miR-206 which is upregulated in mtNSC-34 cells. Previous functional studies on Mctp1 and Rarb have revealed that Mctp1 is related to calcium signaling [30, 31] and that Rarb is associated with cell differentiation [37]. Indeed, we found that the downregulation of Mctp1 and Rarb in mtNSC-34 cells and the intracellular Ca^2+^ levels increased and the neurite outgrowth was reduced.

Previous research introduced us the idea that Mef2c could regulate miR-206 expression [49] and that Hif1α could also induce Mef2c as a transcription factor [48]. In this research, we identified that both Hif1α and Mef2c were increased in mtNSC-34 cells. Earlier studies also shed some light on Hif1α regulation by miR-18b-5p [49]. Importantly, we first discovered that miR-18b-5p expression was significantly decreased in mtNSC-34 cells. Clues pertaining to altered miR-18b-5p, Hif1α, Mef2c, miR-206, Mctp1, and Rarb were perfectly correlated to each other and were related to apoptosis in mtNSC-34 cells. Artificially increased miR-18b-5p directly downregulated Hif1α and Mef2c, restored intracellular Ca^2+^ levels, and induced neuronal differentiation in mtNSC-34 cells, because the decrease in miR-206 caused by reduced Mef2c upregulated both Mctp1 and Rarb in mtNSC-34 cells. In contrast, anti-18b (anti-miR-18b-5p) not only increased Hif1α and Mef2c levels, but also decreased Mctp1 and Rarb. Specifically, anti-18b (anti-miR-18b-5p) induced apoptosis in mNSC and contNSC-34 cells. According to the hypothetical miR-18b-5p signal cascade, serial downregulated Hif1α directly reduced Mef2c, which also decreased miR-206 in mtNSC-34 cells. Mctp1 and Rarb indirectly upregulated by siHif1α decreased apoptosis in mtNSC-34 cells.

We first discovered that Mctp1 and Rarb were involved in the SOD1 mutation and were downregulated by miR-206. Our reporter assay showed that miR-206 significantly decreased both Mctp1 and Rarb, and then inhibited calcium signaling and neuronal differentiation. Increased miR-206 also induced apoptosis in mNSCs and contNSC-34 cells. Indeed, anti-206 (anti-miR-206) recovered apoptosis and increased Mctp1 and Rarb respectively in mtNSC-34 cells. To identify apoptosis by Mctp1 and Rarb, we reduced the levels of Mctp1 and Rarb. Intracellular Ca^2+^ was increased by siMctp1, and neurite outgrowth was decreased by siRarb, but apoptosis was not enhanced. Notably, double-knockdown of Mctp1 and Rarb induced apoptosis in mNSCs and contNSC-34 cells. These results support the hypothesis that Mctp1 and Rarb are simultaneously downregulated by miR-206 and are correlated with apoptosis. This evidence explains that the downregulated miR-18b-5p sequentially regulates Hif1α, Mef2c, miR-206, Mctp1, and Rarb. Other SOD1 mutations (G85R and D90A) also provoked the downregulated miR-18b-5p pathway as G93A does.

We proved that the miR-18b-5p pathway was functional in vitro, but whether or not the downregulated miR-18b-5p pathway could be revealed in the G93A Tg, G86S patient and G17S human MNs was not clear. We also reconfirmed the downregulated miR-18b-5p pathway in the G93A Tg, G86S patient and G17S MNs. As observed in our in vitro studies, miR-18b-5p was incredibly downregulated and miR-206 expression was upregulated in the G93A Tg, G86S patient and G17S MNs. The mRNAs of Hif1α, Mef2c, Mctp1 and Rarb were the same as those observed in vitro studies of the G93A Tg, G86S patient and G17S MNs. Intracellular Ca^2+^ levels were enhanced and MN differentiation was significantly inhibited in G17S MNs. Increased Bax and decreased Bcl2 RNAs also indicated that apoptosis by downregulated miR-18b-5p was elevated in the G93A Tg, G86S patient, and G17S MNs.

According to the recent reports, apoptotic cell death of motor neurons (including SOD1, TDP-43, and FUS) [2–9, 54] and abnormal RNA metabolism (mRNA transcription and miRNAs) [9, 14, 55] in ALS are so controversial issues because non-apoptotic features have been found in ALS patients [54]. Besides, the crucial role of apoptotic cell death by abnormal RNA metabolism is still unclear. We, for the first time, have discovered that downregulated miR-18b-5p, which may be one of the important pathogenic mechanisms in ALS associated SOD1 mutants (D90A, G17S, G85R, G86S and G93A) is associated with the sequential regulation of Hif1α, Mef2c, miR-206, Mctp1, and Rarb. Indeed, downregulated Mctp1 directly increased Ca^2+^ levels, and decreased Rarb significantly reduced cell differentiation in all investigated SOD1 mutations. The causes of down regulated miR-18b-5p by SOD1 mutants need to be further examined. It will provide novel insights into undescribed cellular processes and support to understand that miRNAs are related to important pathogenic mechanisms of sporadic and familial ALS.

## Conclusions

**Fig. 8:**
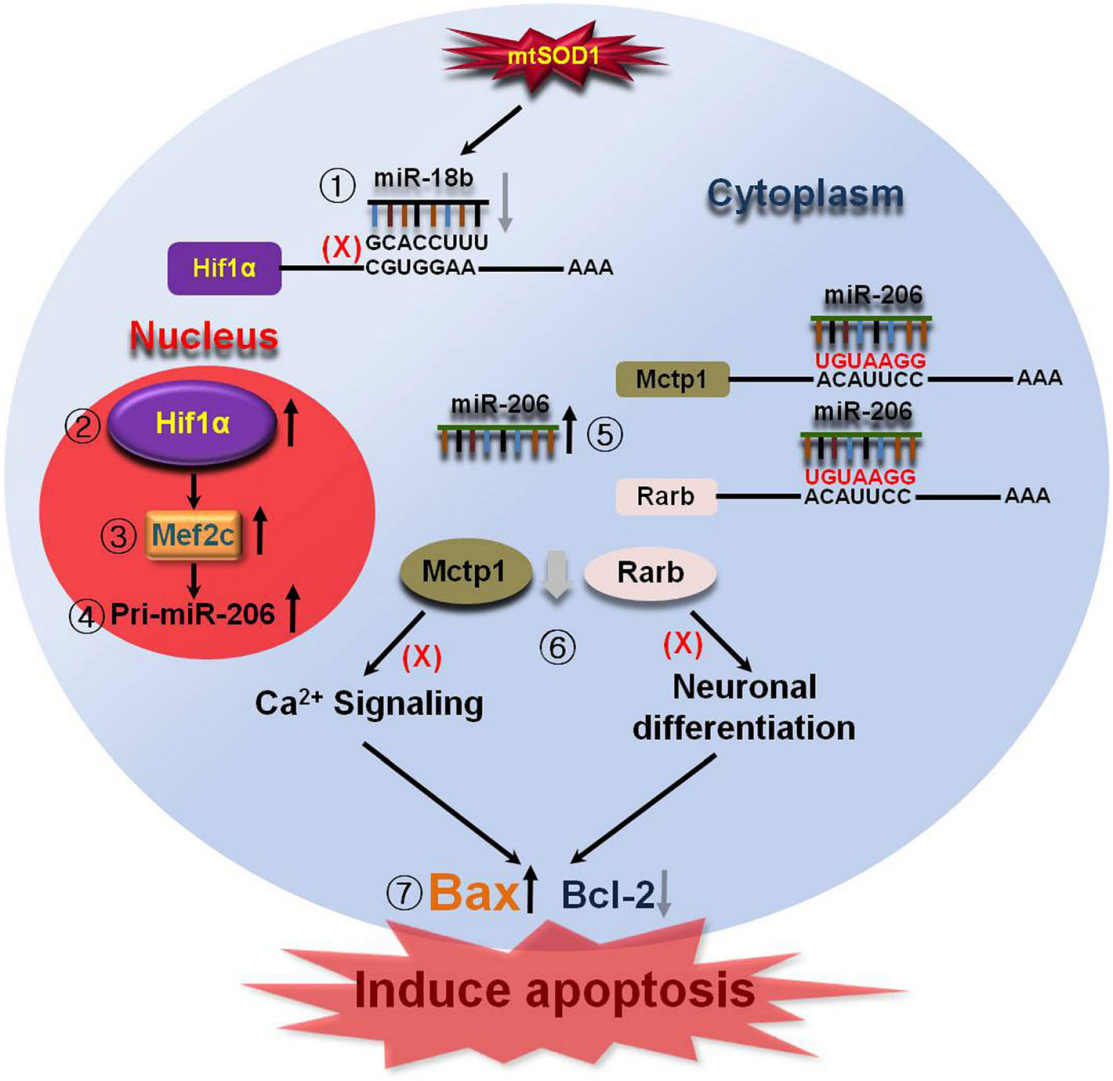
Downregulated miR-18b (miR-18b-5p) signaling pathway in fALS linked SOD1 mutation. A schematic diagram illustrates that downregulated miR-18b (miR-18b-5p) by SOD1 mutation directly increases Hif1α. Mef2c is controlled by overexpressed Hif1α. Increased Mef2c is related with miR-206 expression. Mctp1 and Rarb are downregulated by increased miR-206. Intracellular Ca^2+^ levels is increased by downregulated Mctp1 and Neuronal differentiation is reduced by downregulated Rarb. Apoptotic cell death is induced by prohibited Ca^2+^ signaling and Neuronal differentiation. As a result, downregulated miR-18b-5p by SOD1 mutation leads to apoptotic cell death in fALS linked SOD1 mutation

We have provided strong evidence for the downregulated miR-18b-5p signaling pathway in ALS-related SOD1 mutations (Fig. 8). Our findings are as follows: (i) downregulated miR-18b-5p post-transcriptionally controls Hif1α expression, (ii) Hif1α increases Mef2c as a transcription factor, (iii) Mef2c highly increases miR-206 levels, (iv) miR-206 simultaneously degrades Mctp1 and Rarb, (v) decreased Mctp1 inhibits calcium signaling, (vi) reduced Rarb impedes neuronal cell differentiation, and (vii) downregulated miR-18b-5p enhances apoptotic cell death in ALS SOD1 mutations. This novel mechanism by which the downregulated miR-18b-5p is related to the regulation of several genes (Hif1α, Mef2c, miR-206, Mctp1, and Rarb) requires further investigation before it can be used for clinically meaningful application in ALS treatment.

## Supplementary information

**Additional file 1 Figure S1.** Mctp1 and Rarb are targeted by miR-206 and Hif1α is targeted by miR-18b (miR-18b-5p). (A) A schematic diagram explains consensus base pairing between miR-206 with the 3′ UTR sequences of mouse Mctp1 and Rarb. (B) A schematic diagram shows consensus base pairing between miR-18b (miR-18b-5p) with the 3′ UTR sequences of mouse Hif1α. The identification of miR-18b (miR-18b-5p) target sequences was analyzed by TargetScan (http://www.targetscan.org).

**Additional file 2: Figure S2.** Downregulated miR-18b (miR-18b-5p) by transfected anti-18b (anti-miR-18b-5p) controls alteration of several gene expressions and induces apoptotic cell death in NSC-34 cont cells. (A) anti-18b (anti-miR-18b-5p) increased Hif1α and Mef2c proteins. Both Mctp1 and Rarb proteins were decreased by anti-18b (anti-miR-18b-5p). Upregulated Bax and downregulated Bcl2 by anti-18b (anti-miR-18b-5p) induced apoptotic cell death. (B and C) anti-18b (anti-miR-18b-5p) increased Hif1α and Mef2c transcripts. (D and E) Mctp1 and Rarb mRNAs were decreased by anti-18b (anti-miR-18b-5p). (F and G) Bax mRNAs were upregulated and Bcl2 mRNAs were downregulated under knock down of miR-18b (miR-18b-5p) condition. (H) Lactate dehydrogenase (LDH) release analysis showed that anti-18b (anti-miR-18b-5p) induces cell death. (I and J) RT-qPCR analysis demonstrated decreased miR-18b (miR-18b-5p) and increased miR-206 by anti-18b (anti-miR-18b-5p). (K) Flow cytometry analysis explained that reduced miR-18b (miR-18b-5p) induces apoptotic cell death. Scrambled anti-mir served as a negative control (Cont). The data represent the average ± SEM of 3 separate experiments. Significantly different at *, *p* < 0.05; **, *p* < 0.005.

**Additional file 3: Figure S3.** Knock down of Hif1α reduces apoptotic cell death in mtNSC-34 cells. (A) Transfected siHif1α decreased Mef2c proteins. Mctp1 and Rarb expressions were increased by knock down of Hif1α. siHif1α reduced Bax and induced Bcl2 protein levels. (B and C) RT-qPCR analysis showed downregulated Hif1α by siHif1α decreased Mef2c. (D and E) mRNA levels of Mctp1 and Rarb was increased by siHif1α. (F and G) siHif1α downregulated Bax and upregulated Bcl2 transcripts. (H) miR-206 expression was increased under knock down of Hif1α condition. (I) LDH release analysis showed that siHif1α restored apoptotic cell death. Scrambled siRNA served as a negative control (Cont). Significantly different at *, *p* < 0.05; **, *p* < 0.005. The experiments were replicated 3 times.

**Additional file 4: Figure S4.** Reduced miR-206 in mtNSC-34 cells recovered apoptotic cell death. (A) Western blot analysis showed that transfected anti-206 (anti-miR-206) increased protein levels of Mctp1 and Rarb. Bax protein levels were reduced and Bcl2 protein levels were induced by anti-206 (anti-miR-206), respectively. (B and C) RT-qPCR results showed that Mctp1 and Rarb transcripts also were increased by anti-206 (anti-miR-206). (D and E) Bax mRNAs upregulated and Bcl2 mRNAs downregulated under transfected anti-206 (anti-miR-206) condition, respectively. (F) LDH release assay demonstrated that reduced miR-206 was associated apoptosis. (G) miR-206 was decreased by anti-206 (anti-miR-206). Scrambled anti-mir served as a negative control (Cont). (H and I) Luciferase assay with mutation of miR-206 binding sites (3′ UTR of Mctp1 and Rarb) did not show any significant change. Significantly different at *, *p* < 0.05; **, *p* < 0.005. The experiments were replicated 3 times.

**Additional file 5: Figure S5.** Overexpressed Mctp1 and Rarb reduce apoptotic cell death in mtNSC-34 cells. (A) Co-transfected Mctp1 and Rarb decreased Bax proteins and increased Bcl2 proteins. (B) RT-qPCR analysis explained that mRNA levels of Bax were reduced by cotransfected Mctp1 and Rarb. (C) Bcl2 transcripts were induced by overexpressed Mctp1 and Rarb. (D) LDH release showed that increased Mctp1 and Rarb reduced apoptosis. (E) Transfected Mctp1 reduced intracellular Ca^2+^ levels (Cont (0.025) versus Mctp1 (0.0078) in fluorescence intensities from baseline 490/525 ratio) and RT-qPCR analysis showed increased Mctp1 mRNAs. (F) Overexpressed Rarb enhanced neurite length. Significantly different at *, *p* < 0.05; **, *p* < 0.005. (G) The confocal microscopy presented that overexpressed Rarb (GFP-Rarb) induced neurite outgrowth (MAP2). Empty vector served as a negative control (Cont). Scale bar, 20 μm. The experiments were replicated 5 times.

**Additional file 6: Figure S6.** The apoptotic cell death by SOD1 mutations (G85R and D90A) in fALS is related with miR-18b (miR-18b-5p) signaling pathway. (A) Immunoblot analysis showed that SOD1 (G85R and D90A) mutations increased both Hif1α and Mef2c. Mctp1 and Rarb were decreased by overexpressed SOD1 (G85R and D90A). Increased Bax and decreased Bcl1 proteins by SOD1 (G85R and D90A) were associated with apoptosis in NSC-34 cont cells. (B) RT-qPCR analysis explained that Hif1α and Mef2c were upregulated by SOD1 (G85R and D90A). (C) The mRNA levels of Mctp1 and Rarb was reduced by SOD1 (G85R and D90A). (D) Bax mRNA levels are increased and Bcl2 mRNA levels are decreased under overexpressed SOD1 (G85R and D90A) condition. (E) miR-18b (miR-18b-5p) was reduced by SOD1 (G85R and D90A) (F) miR-206 was upregulated by SOD1 (G85R and D90A). (G) The mRNA levels of SOD1 (G85R and D90A) was increased in NSC-34 cont cells. Empty vector served as a negative control (Cont). The data represent the average ± SEM of 5 separate experiments. Significantly different at *, *p* < 0.05; **, *p* < 0.005.

**Additional file 7: Figure S7.** Downregulated miR-18b (miR-18b-5p) in iPSCs-derived motor neuron from SOD1 (G17S) ALS patient induce apoptotic cell death. (A) RT-PCR analysis verified that hiPSCs from normal and fALS SOD1 (G17S) were generated. (B) The immunoreactivity of ChAT (motor neuron) and MAP2 (neurite outgrowth) was expressed in differentiated motor neurons (normal vs SOD1 (G17S) patient). Scale bar, 20 μm. (C) Bax mRNAs were increased and Bcl2 mRNAs were decreased in iPSCs-derived motor neuron SOD1 (G17S) ALS patient. (D) LDH release analysis demonstrated that the apoptotic cell death was induced in iPSCs-derived motor neuron SOD1 (G17S) ALS patient. Significantly different at *, *p* < 0.05; **, *p* < 0.005. The experiments were replicated 7 times.

**Additional file 8: Table S1** Mouse primer sequences that are used for cloning of 3’UTR of Hif1α, Rarb and Mctp1, miR-18b, miR-206, GFP-Rarb and mCherry-Mctp1. **Table S2** Mouse primer sequences that are used for reverse transcriptase PCR (RT-PCR). **Table S3** Mouse primer sequences that are used for quantitative reverse transcription PCR (RT-qPCR). **Table S4** Human primer sequences that are used for quantitative reverse transcription PCR (RT-qPCR). **Table S5** Human primer sequences that are used for reverse transcriptase PCR (RT-PCR). **Table S6** Human spinal cord and blood samples

## Data Availability

All raw data used and/or analyzed during the current study are available from the corresponding author on reasonable request.

## Abbreviations

**ALS**: Amyotrophic later sclerosis
**SOD1**: Cu/Zn-superoxide dismutase
**FUS**: Fused in Sarcoma
**Hif1α**: Hypoxia inducible factor 1
**iPSCs**: induced pluripotent stem cells
**NSCs**: neural stem cells
**miRNAs**: microRNAs
**miR-18b**: miR-18b-5p
**Mctp1**: Multiple C2 domains transmembrane protein 1
**Mef2c**: Myocyte specific enhancer factor 2c
**MN**: Motor neuron
**Rarb**: Retinoic acid receptor beta
**TDP-43**: TAR DNA-binding protein 43
