# Supplementary material for: Downregulated miR-18b-5p triggers apoptosis by inhibition of calcium signaling and neuronal cell differentiation in transgenic SOD1 (G93A) mice and SOD1 (G17S and G86S) ALS patients
Source: Transl Neurodegener. 2020 Jul 1;9:23. doi: 10.1186/s40035-020-00203-4 (PMC7328278; doi:10.1186/s40035-020-00203-4)
Supplement: Supplementary file 1 — Additional file 1 Figure S1. Mctp1 and Rarb are targeted by miR-206 and Hif1α is targeted by miR-18b (miR-18b-5p). (A) A schematic diagram explains consensus base pairing between miR-206 with the 3′ UTR sequences of mouse Mctp1 and Rarb. (B) A schematic diagram shows consensus base pairing between miR-18b (miR-18b-5p) with the 3′ UTR sequences of mouse Hif1α. The identification of miR-18b (miR-18b-5p) target sequences was analyzed by TargetScan (http://www.targetscan.org). [file 40035_2020_203_MOESM1_ESM.docx]

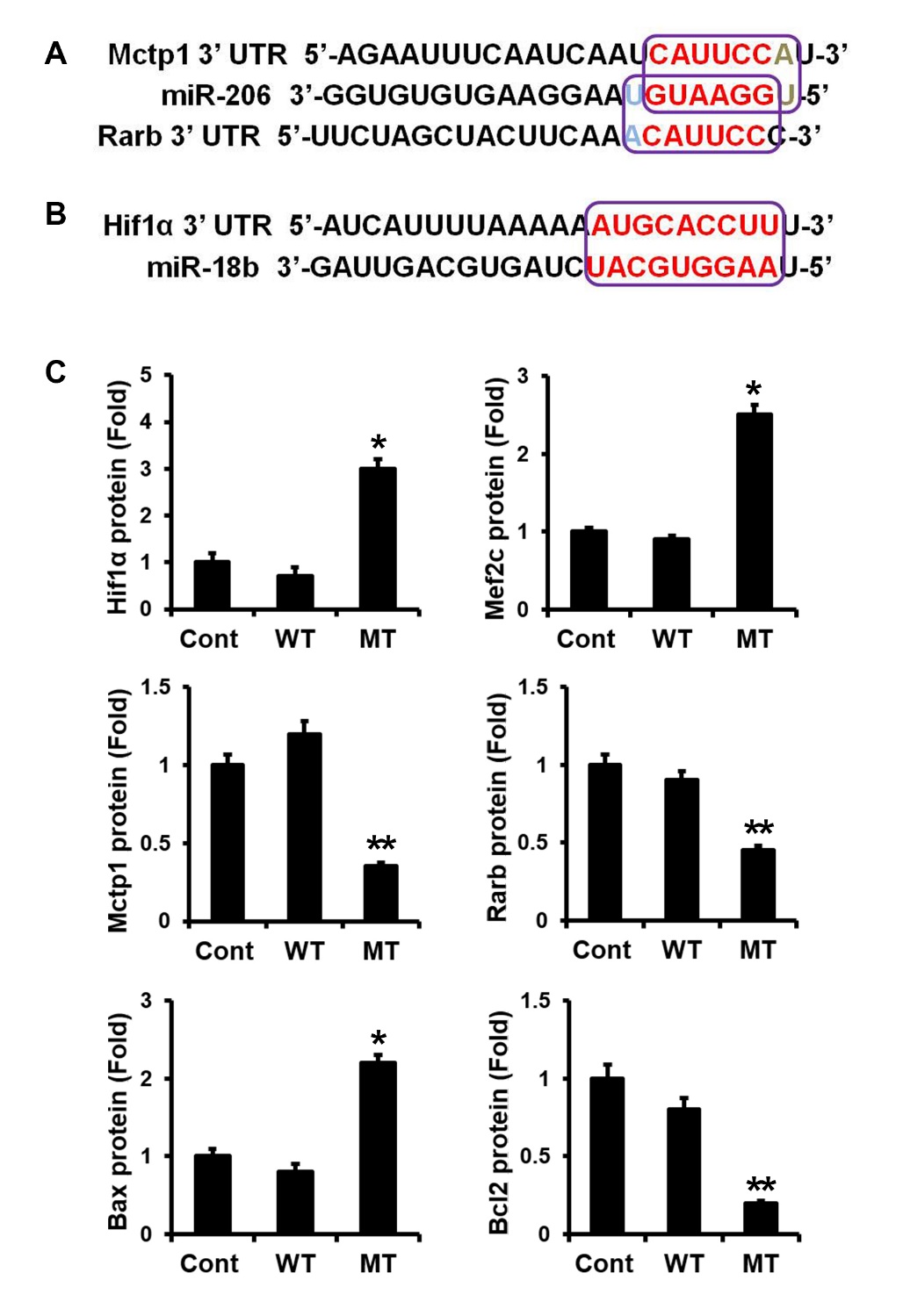


**Figure S1.** Mctp1 and Rarb mRNAs are targeted by miR-206 and Hif1α mRNAs is targeted by miR-18b (miR-18b-5p). (A) A schematic diagram explains consensus base pairing between miR-206 with the 3’ UTR sequences of mouse Mctp1 and Rarb. (B) A schematic diagram shows consensus base pairing between miR-18b (miR-18b-5p) with the 3’ UTR sequences of mouse Hif1α. The identification of miR-18b (miR-18b-5p) target sequences was analyzed by TargetScan (<http://www.targetscan.org>).
