# Supplementary material for: Downregulated miR-18b-5p triggers apoptosis by inhibition of calcium signaling and neuronal cell differentiation in transgenic SOD1 (G93A) mice and SOD1 (G17S and G86S) ALS patients
Source: Transl Neurodegener. 2020 Jul 1;9:23. doi: 10.1186/s40035-020-00203-4 (PMC7328278; doi:10.1186/s40035-020-00203-4)
Supplement: Supplementary file 2 — Additional file 2: Figure S2. Downregulated miR-18b (miR-18b-5p) by transfected anti-18b (anti-miR-18b-5p) controls alteration of several gene expressions and induces apoptotic cell death in NSC-34 cont cells. (A) anti-18b (anti-miR-18b-5p) increased Hif1α and Mef2c proteins. Both Mctp1 and Rarb proteins were decreased by anti-18b (anti-miR-18b-5p). Upregulated Bax and downregulated Bcl2 by anti-18b (anti-miR-18b-5p) induced apoptotic cell death. (B and C) anti-18b (anti-miR-18b-5p) increased Hif1α and Mef2c transcripts. (D and E) Mctp1 and Rarb mRNAs were decreased by anti-18b (anti-miR-18b-5p). (F and G) Bax mRNAs were upregulated and Bcl2 mRNAs were downregulated under knock down of miR-18b (miR-18b-5p) condition. (H) Lactate dehydrogenase (LDH) release analysis showed that anti-18b (anti-miR-18b-5p) induces cell death. (I and J) RT-qPCR analysis demonstrated decreased miR-18b (miR-18b-5p) and increased miR-206 by anti-18b (anti-miR-18b-5p). (K) Flow cytometry analysis explained that reduced miR-18b (miR-18b-5p) induces apoptotic cell death. Scrambled anti-mir served as a negative control (Cont). The data represent the average ± SEM of 3 separate experiments. Significantly different at *, p < 0.05; **, p < 0.005. [file 40035_2020_203_MOESM2_ESM.docx]

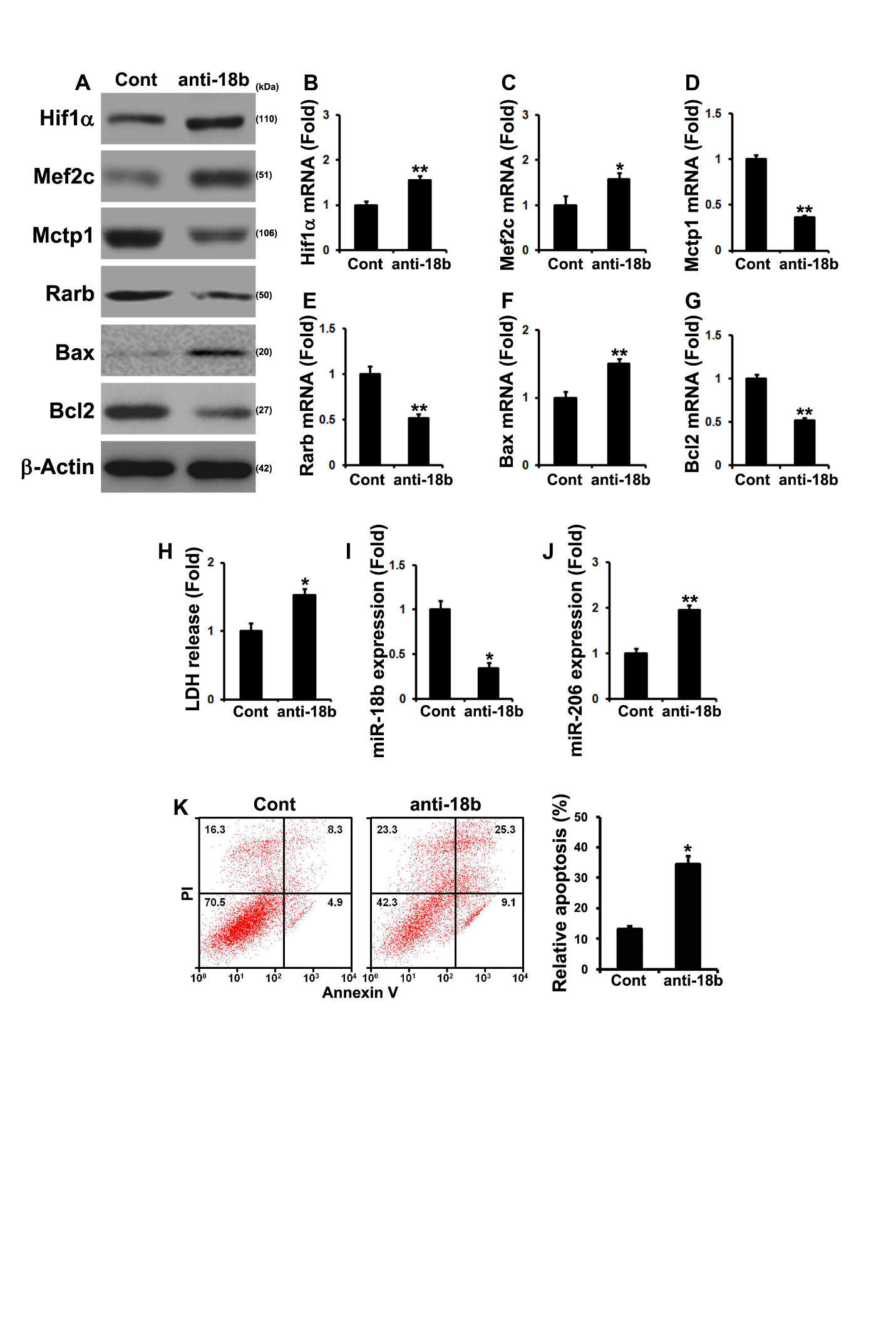


**Figure S2.** Downregulated miR-18b (miR-18b-5p) by anti-18b (anti-miR-18b-5p) controls alteration of several gene expressions and induces apoptotic cell death in NSC-34 cont cells. (A) anti-18b (anti-miR-18b-5p) increased Hif1α and Mef2c proteins. Both Mctp1 and Rarb proteins were decreased by anti-18b (anti-miR-18b-5p). Upregulated Bax and downregulated Bcl2 by anti-18b (anti-miR-18b-5p) induced apoptotic cell death. (B and C) anti-18b (anti-miR-18b-5p) increased Hif1α and Mef2c transcripts. (D and E) Mctp1 and Rarb mRNAs were decreased by anti-18b (anti-miR-18b-5p). (F and G) Bax mRNAs were upregulated and Bcl2 mRNAs were downregulated under knock down of miR-18b (miR-18b-5p) condition. (H) Lactate dehydrogenase (LDH) release analysis showed that anti-18b (anti-miR-18b-5p) induces cell death. (I and J) RT-qPCR analysis demonstrated decreased miR-18b (miR-18b-5p) and increased miR-206 by anti-18b (anti-miR-18b-5p). (K) Flow cytometry analysis explained that reduced miR-18b (miR-18b-5p) induces apoptotic cell death. Scrambled anti-mir served as a negative control (Cont). Fold changes (anti-miR-18b-5p/Cont). The data represent the average ± SEM of 3 separate experiments. Significantly different at *, *p*<0.05; **, *p*<0.005.
