# Supplementary material for: Downregulated miR-18b-5p triggers apoptosis by inhibition of calcium signaling and neuronal cell differentiation in transgenic SOD1 (G93A) mice and SOD1 (G17S and G86S) ALS patients
Source: Transl Neurodegener. 2020 Jul 1;9:23. doi: 10.1186/s40035-020-00203-4 (PMC7328278; doi:10.1186/s40035-020-00203-4)
Supplement: Supplementary file 3 — Additional file 3: Figure S3. Knock down of Hif1α reduces apoptotic cell death in mtNSC-34 cells. (A) Transfected siHif1α decreased Mef2c proteins. Mctp1 and Rarb expressions were increased by knock down of Hif1α. siHif1α reduced Bax and induced Bcl2 protein levels. (B and C) RT-qPCR analysis showed downregulated Hif1α by siHif1α decreased Mef2c. (D and E) mRNA levels of Mctp1 and Rarb was increased by siHif1α. (F and G) siHif1α downregulated Bax and upregulated Bcl2 transcripts. (H) miR-206 expression was increased under knock down of Hif1α condition. (I) LDH release analysis showed that siHif1α restored apoptotic cell death. Scrambled siRNA served as a negative control (Cont). Significantly different at *, p < 0.05; **, p < 0.005. The experiments were replicated 3 times. [file 40035_2020_203_MOESM3_ESM.docx]

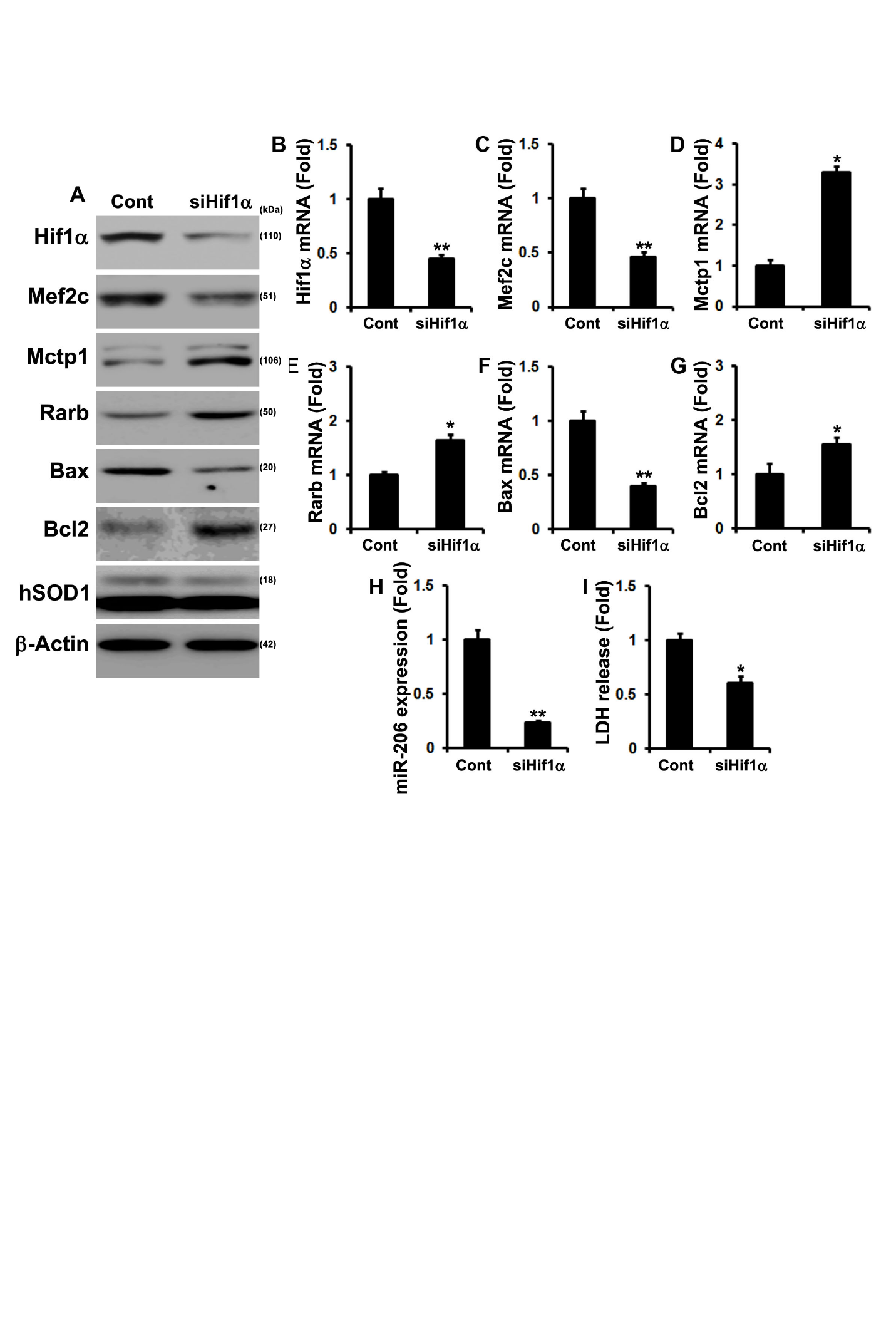


**Figure S3.** Knock down of Hif1α reduces apoptotic cell death in mtNSC-34 cells. (A) Transfected siHif1α decreased Mef2c proteins. Mctp1 and Rarb expressions were increased by knock down of Hif1α. siHif1α reduced Bax and induced Bcl2 protein levels. (B and C) RT-qPCR analysis showed downregulated Hif1α by siHif1α decreased Mef2c. (D and E) mRNA levels of Mctp1 and Rarb was increased by siHif1α. (F and G) siHif1α downregulated Bax and upregulated Bcl2 transcripts. (H) miR-206 expression was increased under knock down of Hif1α condition. (I) LDH release analysis showed that siHif1α restored apoptotic cell death. Fold changes (siHif1α/Cont) Scrambled siRNA served as a negative control (Cont). Significantly different at *, *p*<0.05; **, *p*<0.005. The experiments were replicated 3 times.
