# Supplementary material for: Downregulated miR-18b-5p triggers apoptosis by inhibition of calcium signaling and neuronal cell differentiation in transgenic SOD1 (G93A) mice and SOD1 (G17S and G86S) ALS patients
Source: Transl Neurodegener. 2020 Jul 1;9:23. doi: 10.1186/s40035-020-00203-4 (PMC7328278; doi:10.1186/s40035-020-00203-4)
Supplement: Supplementary file 4 — Additional file 4: Figure S4. Reduced miR-206 in mtNSC-34 cells recovered apoptotic cell death. (A) Western blot analysis showed that transfected anti-206 (anti-miR-206) increased protein levels of Mctp1 and Rarb. Bax protein levels were reduced and Bcl2 protein levels were induced by anti-206 (anti-miR-206), respectively. (B and C) RT-qPCR results showed that Mctp1 and Rarb transcripts also were increased by anti-206 (anti-miR-206). (D and E) Bax mRNAs upregulated and Bcl2 mRNAs downregulated under transfected anti-206 (anti-miR-206) condition, respectively. (F) LDH release assay demonstrated that reduced miR-206 was associated apoptosis. (G) miR-206 was decreased by anti-206 (anti-miR-206). Scrambled anti-mir served as a negative control (Cont). (H and I) Luciferase assay with mutation of miR-206 binding sites (3′ UTR of Mctp1 and Rarb) did not show any significant change. Significantly different at *, p < 0.05; **, p < 0.005. The experiments were replicated 3 times. [file 40035_2020_203_MOESM4_ESM.docx]

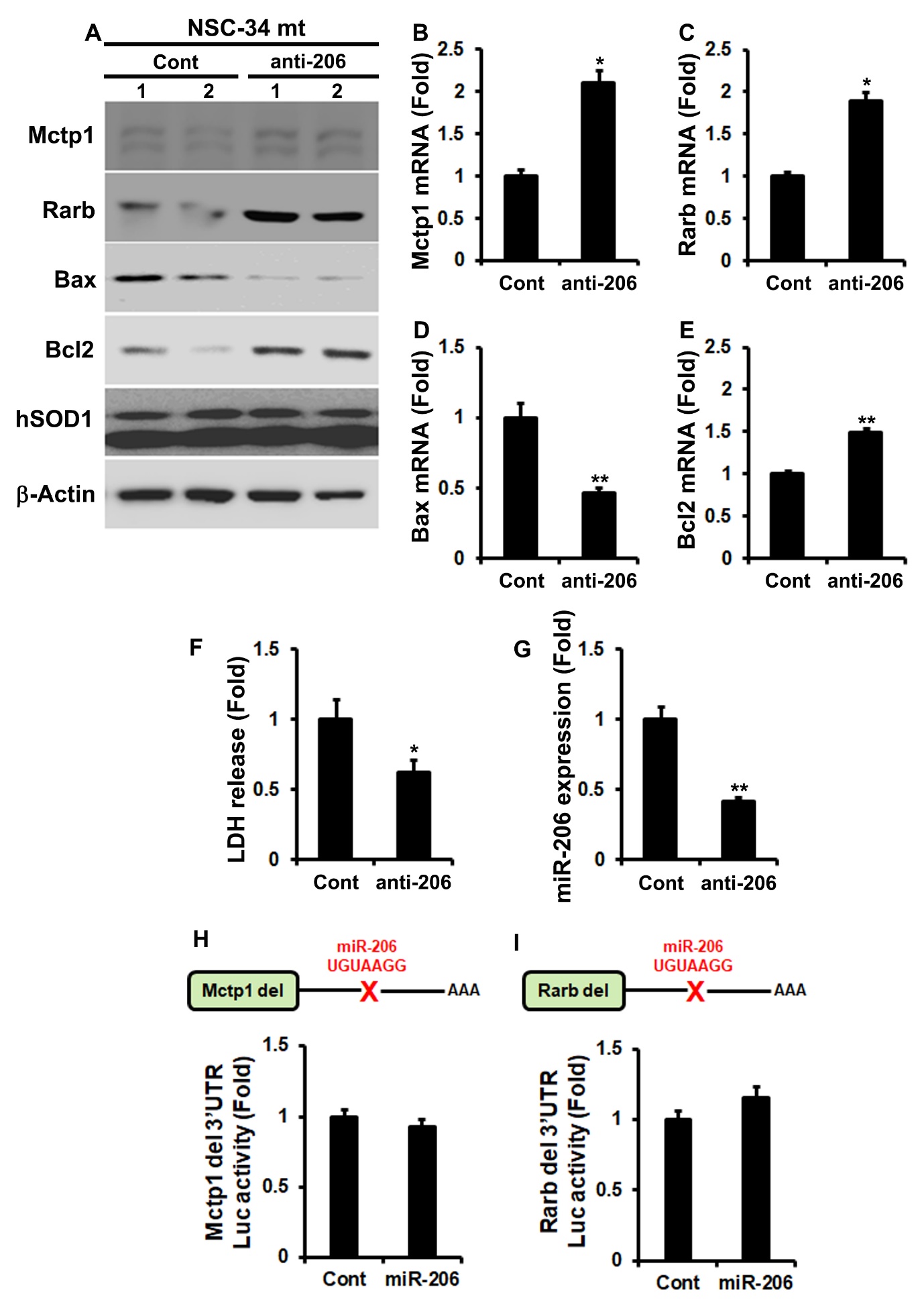


**Figure S4.** Reduced miR-206 in mtNSC-34 cells recovered apoptotic cell death. (A) Western blot analysis showed that transfected anti-206 (anti-miR-206) increased protein levels of Mctp1 and Rarb. Bax protein levels were reduced and Bcl2 protein levels were induced by anti-206 (anti-miR-206), respectively. (B and C) RT-qPCR results showed that Mctp1 and Rarb transcripts also were increased by anti-206 (anti-miR-206). (D and E) Bax mRNAs upregulated and Bcl2 mRNAs downregulated under transfected anti-206 (anti-miR-206) condition, respectively. (F) LDH release assay demonstrated that reduced miR-206 was associated apoptosis. (G) miR-206 was decreased by anti-206. Scrambled anti-mir served as a negative control (Cont). Fold changes (anti-miR-206/Cont) (H and I) Luciferase assay with mutation of miR-206 binding sites (3’ UTR of Mctp1 and Rarb) did not show any significant change. Fold changes (miR-206/Cont) Significantly different at *, *p*<0.05; **, *p*<0.005. The experiments were replicated 3 times.
