# Supplementary material for: Downregulated miR-18b-5p triggers apoptosis by inhibition of calcium signaling and neuronal cell differentiation in transgenic SOD1 (G93A) mice and SOD1 (G17S and G86S) ALS patients
Source: Transl Neurodegener. 2020 Jul 1;9:23. doi: 10.1186/s40035-020-00203-4 (PMC7328278; doi:10.1186/s40035-020-00203-4)
Supplement: Supplementary file 5 — Additional file 5: Figure S5. Overexpressed Mctp1 and Rarb reduce apoptotic cell death in mtNSC-34 cells. (A) Co-transfected Mctp1 and Rarb decreased Bax proteins and increased Bcl2 proteins. (B) RT-qPCR analysis explained that mRNA levels of Bax were reduced by cotransfected Mctp1 and Rarb. (C) Bcl2 transcripts were induced by overexpressed Mctp1 and Rarb. (D) LDH release showed that increased Mctp1 and Rarb reduced apoptosis. (E) Transfected Mctp1 reduced intracellular Ca2+ levels (Cont (0.025) versus Mctp1 (0.0078) in fluorescence intensities from baseline 490/525 ratio) and RT-qPCR analysis showed increased Mctp1 mRNAs. (F) Overexpressed Rarb enhanced neurite length. Significantly different at *, p < 0.05; **, p < 0.005. (G) The confocal microscopy presented that overexpressed Rarb (GFP-Rarb) induced neurite outgrowth (MAP2). Empty vector served as a negative control (Cont). Scale bar, 20 μm. The experiments were replicated 5 times. [file 40035_2020_203_MOESM5_ESM.docx]

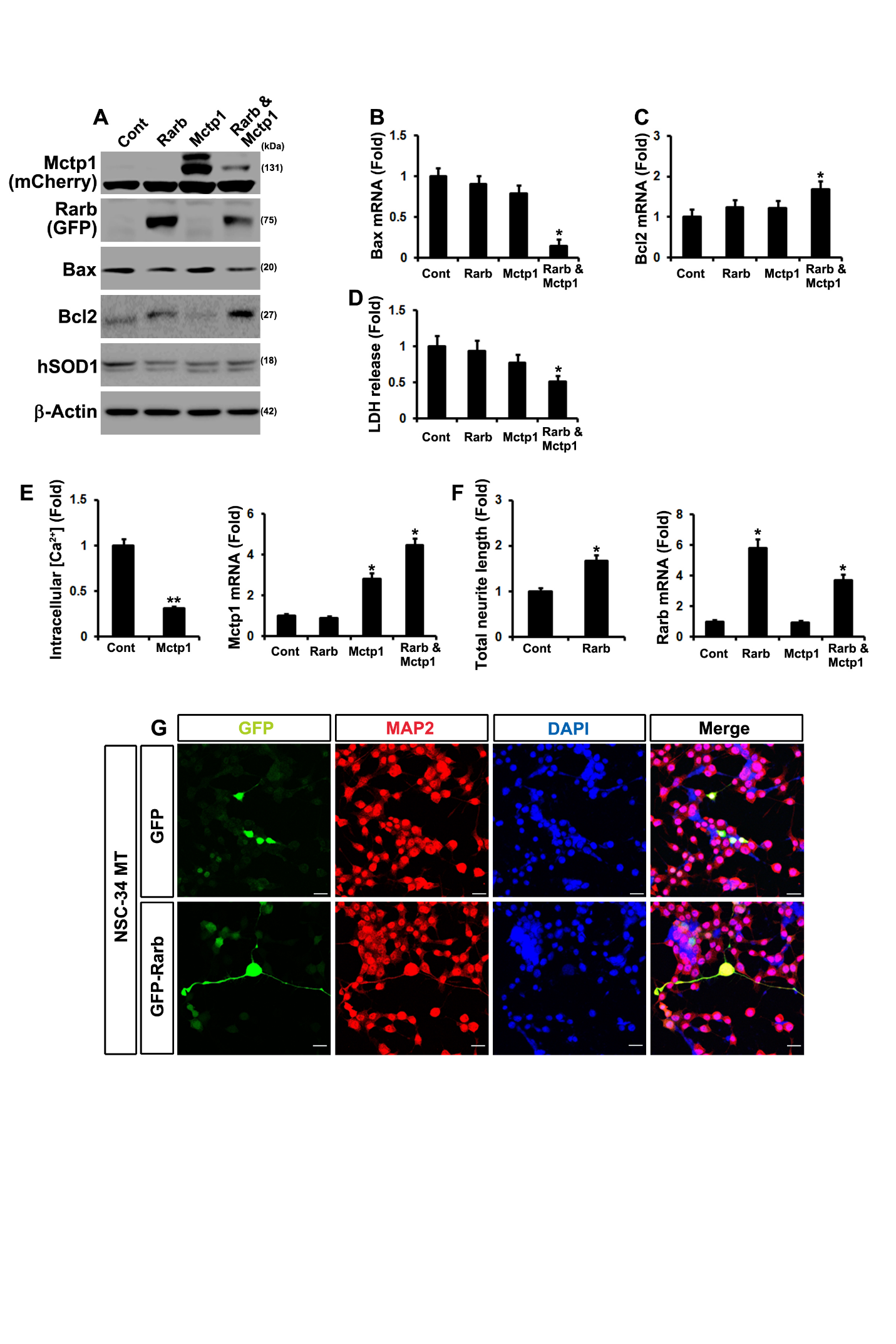


**Figure S5.** Overexpressed Mctp1 and Rarb reduce apoptotic cell death in mtNSC-34 cells. (A) Co-transfected Mctp1 and Rarb decreased Bax proteins and increased Bcl2 proteins. (B) RT-qPCR analysis explained that mRNA levels of Bax were reduced by cotransfected Mctp1 and Rarb. (C) Bcl2 transcripts were induced by overexpressed Mctp1 and Rarb. (D) LDH release showed that increased Mctp1 and Rarb reduced apoptosis. (E) Transfected Mctp1 reduced intracellular Ca^2+^ levels (Cont (0.025) vs Mctp1 (0.0078) in fluorescence intensities from baseline 490/525 ratio) and RT-qPCR analysis showed increased Mctp1 mRNAs. (F) Overexpressed Rarb enhanced neurite length. Fold changes (Rarb/Cont, Mctp1/Cont and (Rarb & Mctp1)/Cont). Significantly different at *, *p*<0.05; **, *p*<0.005. (G) The confocal microscopy presented that overexpressed Rarb (GFP-Rarb) induced neurite outgrowth (MAP2). Empty vector served as a negative control (Cont). Scale bar, 20 µm. The experiments were replicated 5 times.
