# Supplementary material for: Downregulated miR-18b-5p triggers apoptosis by inhibition of calcium signaling and neuronal cell differentiation in transgenic SOD1 (G93A) mice and SOD1 (G17S and G86S) ALS patients
Source: Transl Neurodegener. 2020 Jul 1;9:23. doi: 10.1186/s40035-020-00203-4 (PMC7328278; doi:10.1186/s40035-020-00203-4)
Supplement: Supplementary file 6 — Additional file 6: Figure S6. The apoptotic cell death by SOD1 mutations (G85R and D90A) in fALS is related with miR-18b (miR-18b-5p) signaling pathway. (A) Immunoblot analysis showed that SOD1 (G85R and D90A) mutations increased both Hif1α and Mef2c. Mctp1 and Rarb were decreased by overexpressed SOD1 (G85R and D90A). Increased Bax and decreased Bcl1 proteins by SOD1 (G85R and D90A) were associated with apoptosis in NSC-34 cont cells. (B) RT-qPCR analysis explained that Hif1α and Mef2c were upregulated by SOD1 (G85R and D90A). (C) The mRNA levels of Mctp1 and Rarb was reduced by SOD1 (G85R and D90A). (D) Bax mRNA levels are increased and Bcl2 mRNA levels are decreased under overexpressed SOD1 (G85R and D90A) condition. (E) miR-18b (miR-18b-5p) was reduced by SOD1 (G85R and D90A) (F) miR-206 was upregulated by SOD1 (G85R and D90A). (G) The mRNA levels of SOD1 (G85R and D90A) was increased in NSC-34 cont cells. Empty vector served as a negative control (Cont). The data represent the average ± SEM of 5 separate experiments. Significantly different at *, p < 0.05; **, p < 0.005. [file 40035_2020_203_MOESM6_ESM.docx]

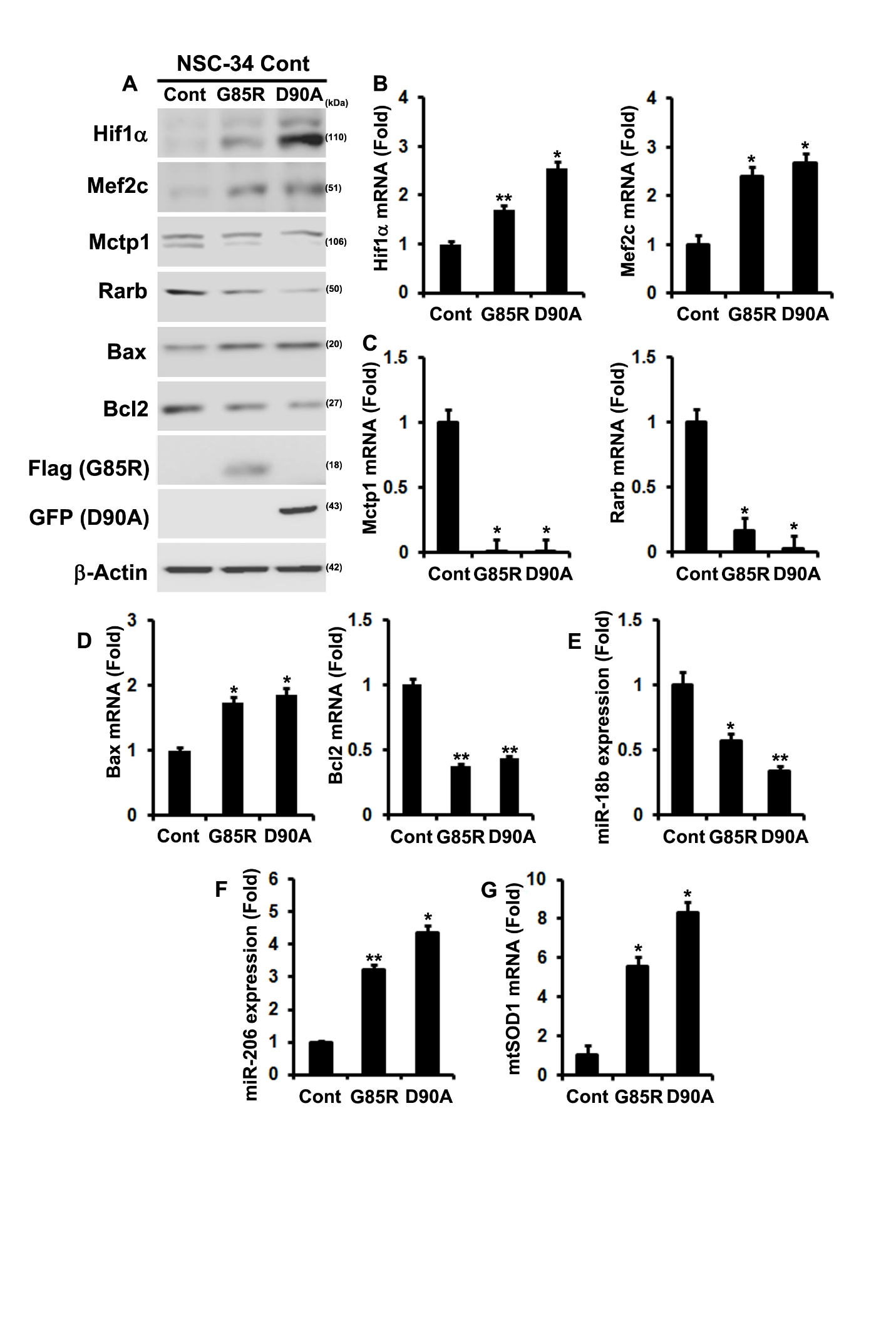


**Figure S6.** The apoptotic cell death by SOD1 mutations (G85R and D90A) in fALS is related with miR-18b (miR-18b-5p) signaling pathway. (A) Immunoblot analysis showed that SOD1 (G85R and D90A) mutations increased both Hif1α and Mef2c. Mctp1 and Rarb were decreased by overexpressed SOD1 (G85R and D90A). Increased Bax and decreased Bcl1 proteins by SOD1 (G85R and D90A) were associated with apoptosis in NSC-34 cont cells. (B) RT-qPCR analysis explained that Hif1α and Mef2c were upregulated by SOD1 (G85R and D90A). (C) The mRNA levels of Mctp1 and Rarb was reduced by SOD1 (G85R and D90A). (D) Bax mRNA levels are increased and Bcl2 mRNA levels are decreased under overexpressed SOD1 (G85R and D90A) condition. (E) miR-18b (miR-18b-5p) was reduced by SOD1 (G85R and D90A) (F) miR-206 was upregulated by SOD1 (G85R and D90A). (G) The mRNA levels of SOD1 (G85R and D90A) was increased in NSC-34 cont cells. Empty vector served as a negative control (Cont). The data represent the average ± SEM of 5 separate experiments. Fold changes (G85R/Cont and D90A/Cont). Significantly different at *, *p*<0.05; **, *p*<0.005.
