# Supplementary material for: Downregulated miR-18b-5p triggers apoptosis by inhibition of calcium signaling and neuronal cell differentiation in transgenic SOD1 (G93A) mice and SOD1 (G17S and G86S) ALS patients
Source: Transl Neurodegener. 2020 Jul 1;9:23. doi: 10.1186/s40035-020-00203-4 (PMC7328278; doi:10.1186/s40035-020-00203-4)
Supplement: Supplementary file 7 — Additional file 7: Figure S7. Downregulated miR-18b (miR-18b-5p) in iPSCs-derived motor neuron from SOD1 (G17S) ALS patient induce apoptotic cell death. (A) RT-PCR analysis verified that hiPSCs from normal and fALS SOD1 (G17S) were generated. (B) The immunoreactivity of ChAT (motor neuron) and MAP2 (neurite outgrowth) was expressed in differentiated motor neurons (normal vs SOD1 (G17S) patient). Scale bar, 20 μm. (C) Bax mRNAs were increased and Bcl2 mRNAs were decreased in iPSCs-derived motor neuron SOD1 (G17S) ALS patient. (D) LDH release analysis demonstrated that the apoptotic cell death was induced in iPSCs-derived motor neuron SOD1 (G17S) ALS patient. Significantly different at *, p < 0.05; **, p < 0.005. The experiments were replicated 7 times. [file 40035_2020_203_MOESM7_ESM.docx]

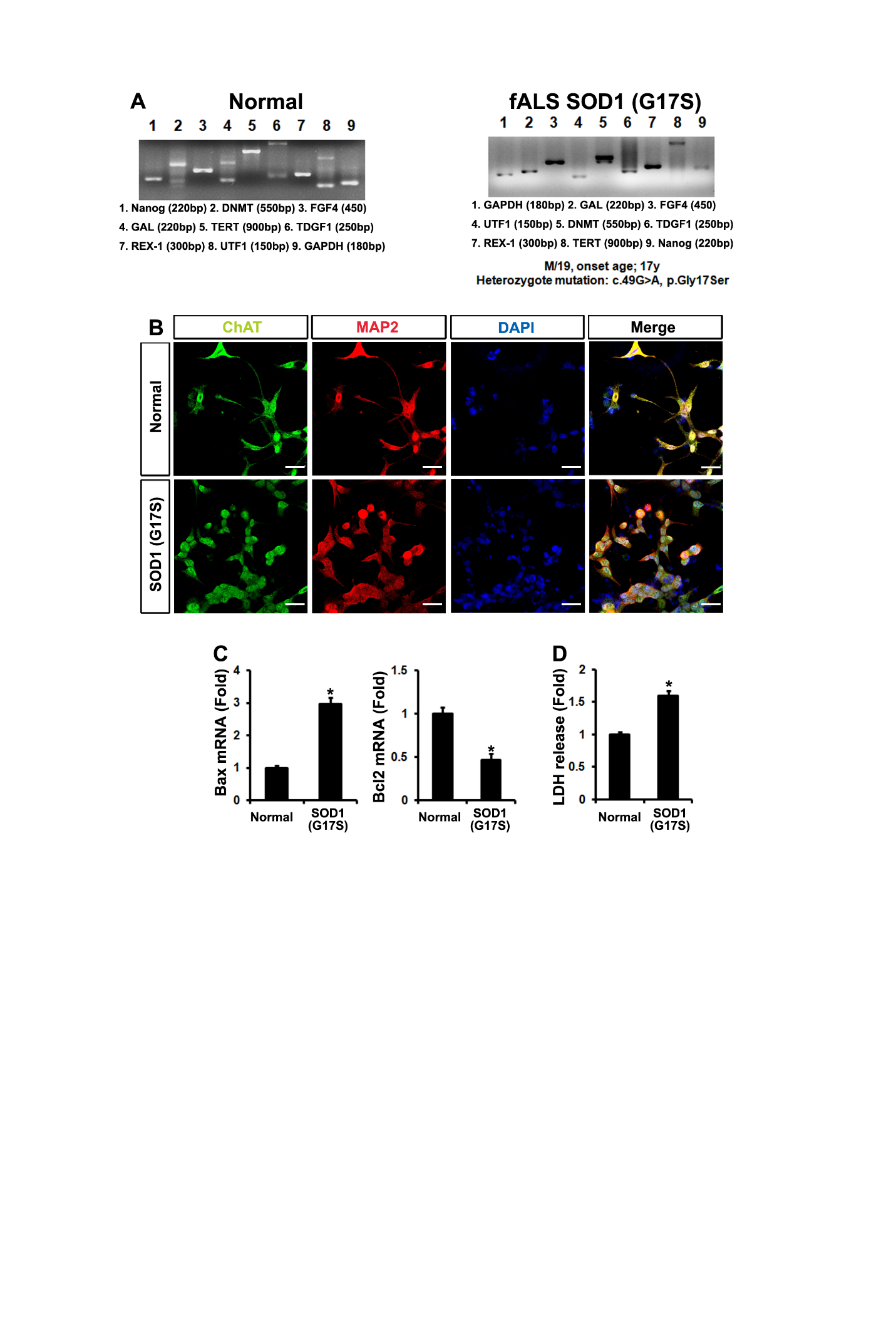


**Figure S7.** Downregulated miR-18b (miR-18b-5p) in iPSCs-derived motor neuron from SOD1 (G17S) ALS patient induce apoptotic cell death. (A) RT-PCR analysis verified that hiPSCs from normal and fALS SOD1 (G17S) were generated. (B) The immunoreactivity of ChAT (motor neuron) and MAP2 (neurite outgrowth) was expressed in differentiated motor neurons (normal vs SOD1 (G17S) patient). Scale bar, 20 µm. (C) Bax mRNAs were increased and Bcl2 mRNAs were decreased in iPSCs-derived motor neuron SOD1 (G17S) ALS patient. (D) LDH release analysis demonstrated that the apoptotic cell death was induced in iPSCs-derived motor neuron SOD1 (G17S) ALS patient. Fold changes (SOD1(G17S)/Normal). Significantly different at *, *p*<0.05; **, *p*<0.005. The experiments were replicated 7 times.
