# Supplementary material for: Downregulated miR-18b-5p triggers apoptosis by inhibition of calcium signaling and neuronal cell differentiation in transgenic SOD1 (G93A) mice and SOD1 (G17S and G86S) ALS patients
Source: Transl Neurodegener. 2020 Jul 1;9:23. doi: 10.1186/s40035-020-00203-4 (PMC7328278; doi:10.1186/s40035-020-00203-4)
Supplement: Supplementary file 8 — Additional file 8: Table S1 Mouse primer sequences that are used for cloning of 3’UTR of Hif1α, Rarb and Mctp1, miR-18b, miR-206, GFP-Rarb and mCherry-Mctp1. Table S2 Mouse primer sequences that are used for reverse transcriptase PCR (RT-PCR). Table S3 Mouse primer sequences that are used for quantitative reverse transcription PCR (RT-qPCR). Table S4 Human primer sequences that are used for quantitative reverse transcription PCR (RT-qPCR). Table S5 Human primer sequences that are used for reverse transcriptase PCR (RT-PCR). Table S6 Human spinal cord and blood samples [file 40035_2020_203_MOESM8_ESM.docx]

**Table S1** Mouse primer sequences that are used for cloning of 3’UTR of Hif1α, Rarb and Mctp1, miR-18b, miR-206, GFP-Rarb and mCherry-Mctp1.

| Gene | Sequence |
| --- | --- |
| Hif1α 3’UTR | F- CCGCTCGAGTGTTGGTTATTTTTGGACACT  R- CTAGTCTAGAATATTGCATGAGTAACTGCTGGT |
| Rarb 3’UTR | F- CCGCTCGAGAACGTGTAATTACCTTGAAA  R- CTAGTCTAGACAAAGTCTTCAGAAACTTAA |
| Mctp1 3’UTR | F- CCGCTCGAGAAAGCTTGAATAATAGAAAT  R- CTAGTCTAGAATACATGGGTTTTTTGTTTG |
| miR-18b | F- CGCGGATCCACCATGGTGATTTAATCAGA  R- CCGCTCGAGCCGTTCAAATCATTTCTCAA |
| miR-206 | F- CGCGGATCCATTCTTCACACTTCTCACTT  R- CCGCTCGAG ACGAAGAAGTCAACAGCATA |
| GFP-Rarb | F- CCGCTAGCATGAGCACCAGCAGCCACGC  R- CCACCGGTCTGCAGCAGTGGTGACTGAC |
| mCherry-Mctp1 | F- CCCAAGCTTATGTACCAGTTGGATATCACACTA R- CCCAAGCTTGCCAAGGTTGTTTTTTCTTCC |
| Mctp1 del 3’UTR | F- CTAAAACCTTAAAATCTCTT  R- ATAGAAGGCTTAAAAGTCTT |
| Rarb del 3’UTR | F- AGGACCTTCAGTTCCAGATT  R- CTGCAACAGCTGGAAATGTC |

**Table S2** Mouse primer sequences that are used for reverse transcriptase PCR (RT-PCR).

| Gene | Sequence |
| --- | --- |
| Mctp1 intron | F- GACTCCAACATACCCATTTCTG  R- TAATATCTCTTCCCGCTCCTTC |
| Mctp1 exon | F- TCATCCTTACGCCTAAGGAAG  R- CCGGAACTTCACATATGGATC |
| Rarb intron | F- CACCTGAAGGTGAATGTTGG  R- CACTTGAACTTGGGGTCAAG |
| Rarb exon | F- GATCTACACTTGCCATCGAGA  R- CTTTCCGGATCTTCTCAGTGA |
| GAPDH intron | F- TGGTGCAACAGTATTCCACT  R- CTGGAACATGTAGACCATGTAG |
| GAPDH exon | F- CATGTTTGTGATGGGTGTGA  R- GATGCAGGGATGATGTTCTG |

**Table S3** Mouse primer sequences that are used for quantitative reverse transcription PCR (RT-qPCR).

| Gene | Sequence |
| --- | --- |
| Hif1α | F- GTTCACCAAAGTTGAATCAGAGG  R- CGATGAAGGTAAAGGAGACATTG |
| Mef2c | F- AGGATAATGGATGAGCGTAACAG  R- AGCAACACCTTATCCATGTCAGT |
| Mctp1 | F- CGTTGTGTCATAGTGCTTGTAAA  R- ATCATGTAGAGCTCAAAGTTCCA |
| Rarb | F- TTTCTCTGATGGCCTTACACTAA  R- AGATTAAACAGATGGCACTGAGA |
| Bax | F- AAGCTGAGCGAGTGTCTCCG  R- GGAGGAAGTCCAGTGTCCAG |
| Bcl2 | F- AACCCAATGCCCGCTGTGCA  R- ACCGAACTCAAAGAAGGCCACAA |
| GAPDH | F- ATAGCTGATGGCTGCAGGTT  R- AATCTCCACTTTGCCACTGC |

**Table S4** Human primer sequences that are used for quantitative reverse transcription PCR (RT-qPCR).

| Gene | Sequence |
| --- | --- |
| Hif1α | F- AGATAGCAAGACTTTCCTCAGTC  R- CTGTGGTGACTTGTCCTTTAGTA |
| Mef2c | F- CTACTTTACCAGGACAAGGAATG  R- CTGAGATAAATGAGTGCTAGTGC |
| Mctp1 | F- AGGAATAGTCAGCATCACCTTGA  R- CAATGACTCCTCCTCTTTCTTCA |
| Rarb | F- CTTCTCAGTGCCATCTGCTTAAT  R- AATTACACGCTCTGCACCTTTAG |
| Bax | F- AAGCTGAGCGAGTGTCTCAA  R- AGTAGAAAAGGGCGACAACC |
| Bcl2 | F- ACGCCCCATCCAGCCGCATC  R- CACACATGACCCCACCGAACTCA |
| GAPDH | F- CTGCATTCGCCCTCTTAATG  R- TGAGGTCAATGAAGGGGTCA |
| SOD1 | F- GTGGGGAAGCATTAAAGGACTGAC  R- CAATTACACCACAAGCCAAACGAC |

**Table S5** Human primer sequences that are used for reverse transcriptase PCR (RT-PCR).

| Gene | Sequence |
| --- | --- |
| Nanog | F- TTCCTTCCTCCATGGATCTG  R- TGCTGGAGGCTGAGGTCTTT |
| DNMT | F- TGCTGCTCACAGGGCCCGATACTTC  R- TCCTTTCGAGCTCAGTGCACCACAAAAC |
| FGF4 | F- CTACAACGCCTACGAGTCCTACA  R- GTTGCACCAGAAAAGTCAGAGTTG |
| GAL | F- TGCGGCCCGAAGATCACATGAAACC  R- CCCAGGAGGCTCTCAGGACCGCTC |
| TERT | F- CCTGCTCAAGCTGACTCGACACCGTG  R- GGAAAAGCTGGCCCTGGGGTGGAGC |
| TDGF1 | F- CTGCTGCCTGAARGGGGGAACCTGC  R- GCCACGAGGTGCTCATCCATCACAAGG |
| REX-1 | F- CAGATCCTAAACAGCTCGCAGAAT  R- GCGTACGCAAATTAAAGTCCAGA |
| UTF1 | F- CCGTCGCTGAACACCGCCCTGCTG  R- CGCGCTGCCCAGAATGAAGCCCAC |
| GAPDH | F- AGAAGGCTGGGGCTCATTTG  R- AGGGGCCATCCACAGTCTTC |

**Table S6** Human spinal cord and blood samples.

| Subjects | Diagnosis | Spinal cord region | Age at sampling | Gender | Methods |
| --- | --- | --- | --- | --- | --- |
| Control 1 | Non-demented control | Cervical | 73 | F | Western blot and RT-qPCRs |
| Control 2 | Non-demented control | Cervical | 66 | M | Western blot and RT-qPCRs |
| Control 3 | Non-demented control | Blood | 52 | M | iPSCs-derived motor neuron |
| FALS Patient 1 | SOD1 (G86S) | Cervical, Lumber | 57 | M | Western blot and RT-qPCRs |
| FALS Patient 2 | SOD1 (G17S) | Blood | 24 | M | iPSCs-derived motor neuron |
